# Supplementary material for: Total Synthesis of Nicrophorusamide A and Structural Disproof of the Proposed Noursamycin A
Source: Molecules. 2023 Nov 6;28(21):7442. doi: 10.3390/molecules28217442 (PMC10647733; doi:10.3390/molecules28217442)
Supplement: Supplementary file 1 [file molecules-28-07442-s001.zip › molecules-2657698-supplementary.pdf]

Supplementary Information

Table of Contents:

1. Optimization of Macrocyclization. S2

2. Synthetic Schemes of Compounds **19**, **20**, **22**, **24**. S3

3. NMR Data Comparison between Natural Noursamycin A and Synthetic Sample **1**. S4

4. NMR Data Comparison between Natural Nicrophorusamide A and Synthetic Sample **25**. S7

5. NMR Spectra. S10

6. References. S30

1. Optimization of Macrocyclization.

Table S1. Optimization of macrocyclization of linear precursor.

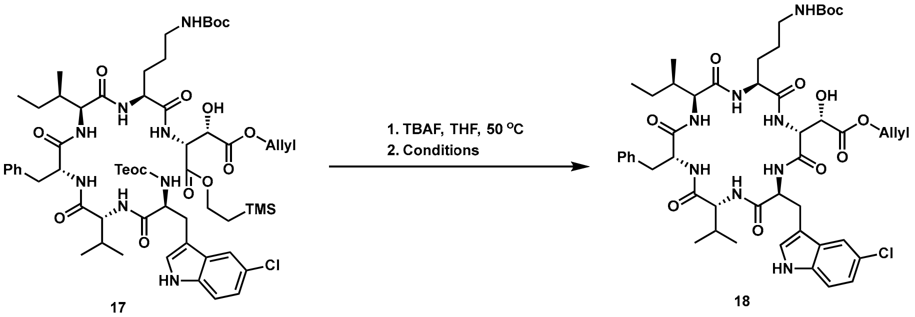

17 18

Entry	Conditions (equiv)	Yield (%) over 2 steps
1	HATU (1.5), HOAt (1.5), DIPEA (2.0), DMF	35
2	BEP (3.0), DIPEA (6.0), DCM	24
3	EDCI (2.0), HOAt (2.0), DMAP (0.2), DCM	58
4	PyAOP (3.0), DIPEA (6.0), DCM	42
5	FDPP (3.0), DIPEA (6.0), DCM	trace
6	EDCI (2.0), HOAt (2.0), DMAP (0.2), DCM:DMF=20:1	65

2. Synthetic Schemes of Compounds **19**, **20**, **22**, **24**.

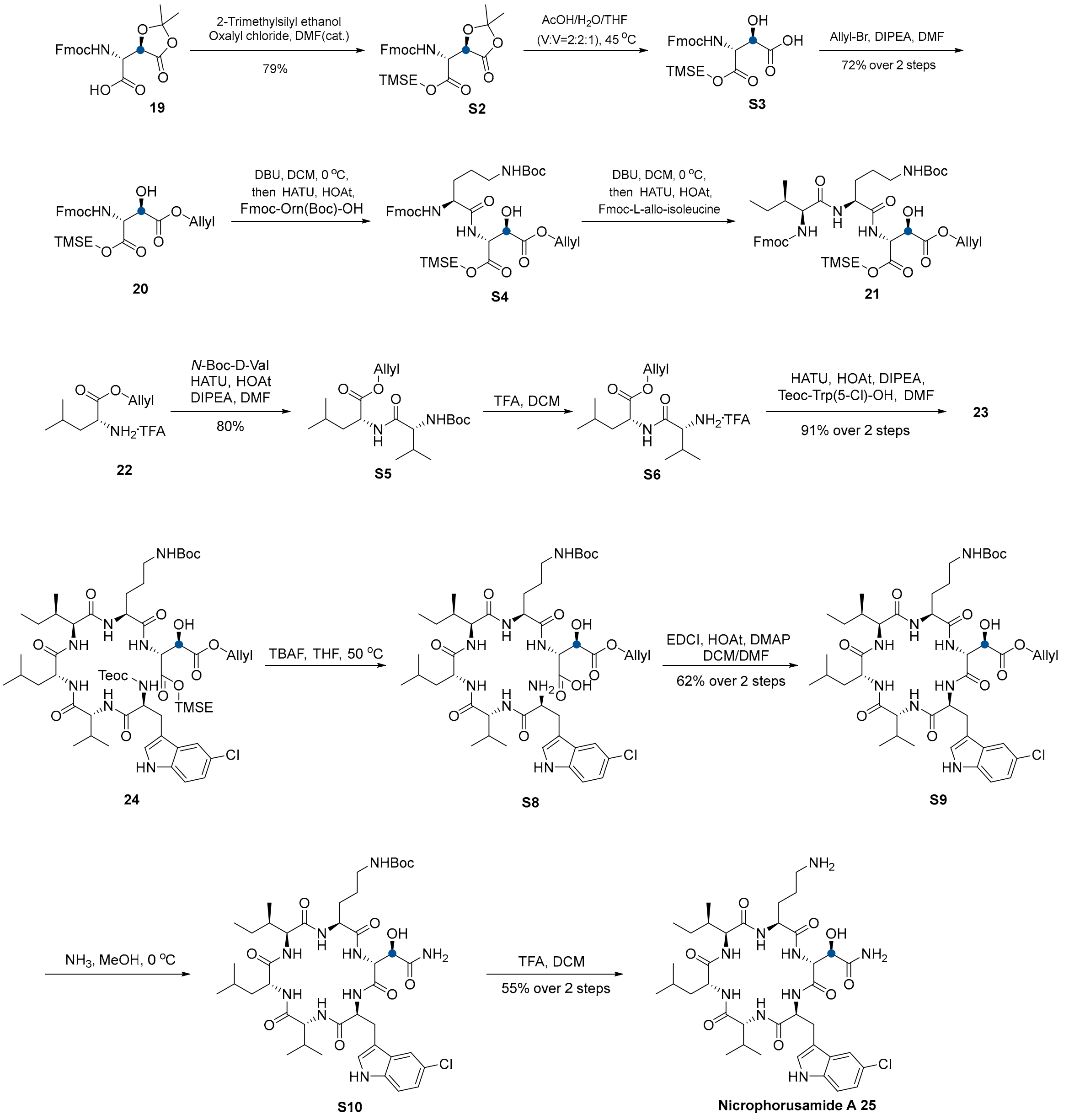

3. NMR Data Comparison between Natural Noursamycin A and Synthetic Sample **1**.

Table S2. ^{13}C NMR[a] comparison of natural noursamycin A and synthetic sample **1**.

Amino acid Residue	Noursamycin A			
	Pos.	Natural (δ_{A1})	Synthetic Sample **1** (δ_{A2})	$\Delta_{\delta} = \delta_{A1}-\delta_{A2}$
Trp[1] 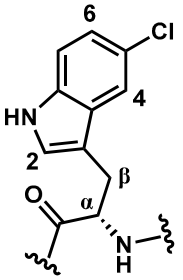	α	54.1	52.6	1.5
	β	27.5	27.7	-0.2
	2	126.0	125.6	0.4
	3	110.3	110.1	0.2
	3a	128.8	128.3	0.5
	4	118.0	117.3	0.7
	5	123.4	123.0	0.4
	6	121.2	120.7	0.5
	7	113.2	112.8	0.5
	7a	135.1	134.5	0.6
	COOH	171.7	171.1	0.6
Val[2] 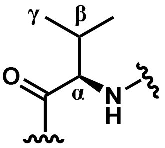	α	60.9	60.6	0.3
	β	29.5	29.7	-0.2
	γ_1	19.3	19.1	0.2
	γ_2	19.1	18.8	0.3
	COOH	171.4	170.6	0.8
Phe[3] 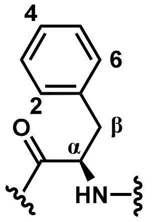	α	54.9	53.9	1.0
	β	37.4	38.5	-1.1
	1	138.4	137.2	1.2
	2, 6	129.3	129.3	0.0
	3, 5	128.4	128.1	0.3
	4	126.6	126.3	0.3

	COOH	171.6	170.7	0.9
Ile[4] 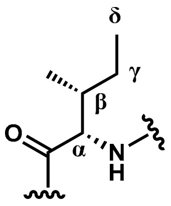	α	57.5	58.2	-0.7
	β	36.7	35.8	0.9
	γ(methyl)	14.8	14.3	0.5
	γ	26.0	25.4	0.6
	δ	11.1	11.6	-0.5
	COOH	172.2	171.4	0.8
Orn[5] 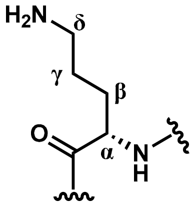	NH2	-	-	-
	α	53.6	52.4	1.2
	β	27.5	26.1	1.4
	γ	25.5	24.2	1.3
	δ	39.5	overlap	-
	COOH	172.5	172.1	0.4
OH-ASN[6] 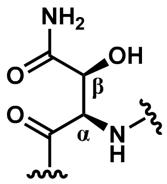	α	56.1	56.6	-0.5
	β	71.6	70.6	1.0
	CO-	173.8	174.2	-0.4
	COOH	170.0	169.0	1.0

[a]All chemical shifts are reported in ppm. All spectra were measured in DMSO-d_6 and referenced to the residual solvent peak at δ_H = 2.50 ppm and δ_C = 39.52 ppm as done in the original isolation report.[1] ^{13}C-NMR spectrum was recorded at 126 MHz.

Figure S1. Comparison of ^{1}H NMR (DMSO-d_6, 500 MHz) spectra of synthetic sample **1** (red) and noursamycin A (blue, screenshot from supporting information of noursamycin A's isolation article published in 2019[1]).

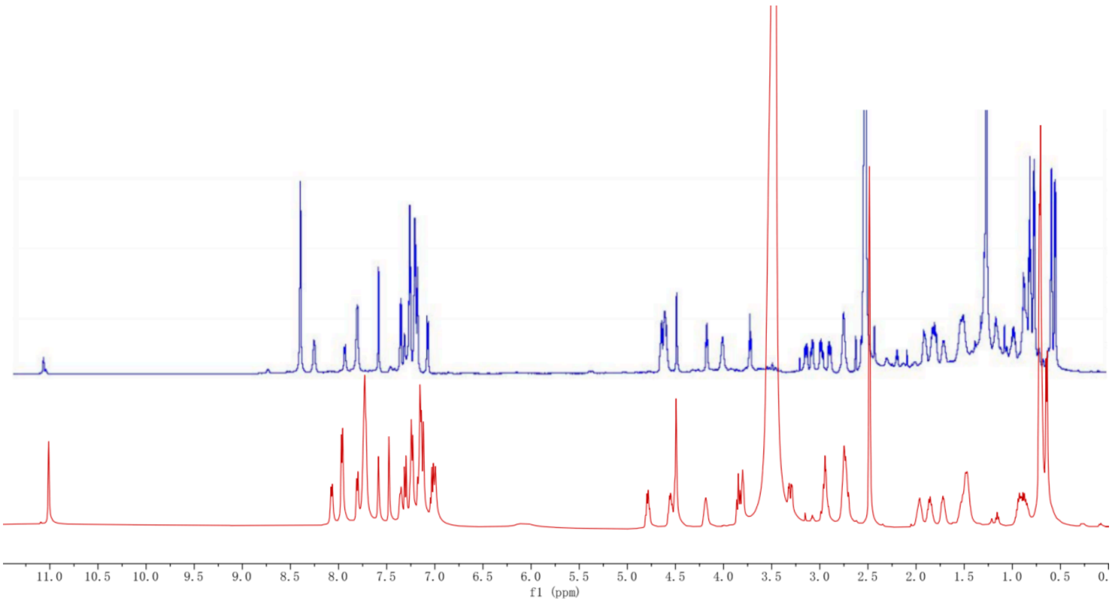

4. NMR Data Comparison between Natural Nicrophorusamide A and Synthetic Sample **25**.

Table S3. NMR[a] comparison between natural nicrophorusamide A and synthetic sample **25**.

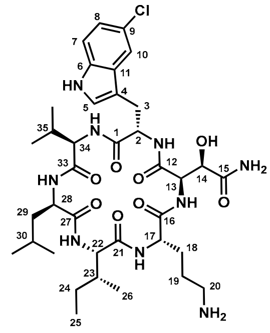

Nicrophorusamide A

Carbon No.	^{13}C NMR		$\Delta\delta^{13}$C
	natural / δ_{A1} (ppm)	synthetic / δ_{A2} (ppm)	δ_{A1}-δ_{A2}
1	171.1	171.1	0.0
2	53.5	53.5	0.0
3	26.7	26.7	0.0
4	109.8	109.8	0.0
5	125.4	125.5	-0.1
6	134.5	134.5	0.0
7	112.7	112.7	0.0
8	120.7	120.7	0.0
9	122.9	123.0	-0.1
10	117.5	117.5	0.0
11	128.4	128.4	0.0
12	169.4	169.5	-0.1
13	55.8	55.7	0.1
14	70.8	70.8	0.0
15	173.2	173.2	0.0
16	170.6	170.6	0.0

17	53.1	53.2	-0.1
18	26.8	26.8	0.0
19	23.7	23.8	-0.1
20	38.4	38.4	0.0
21	171.7	171.7	0.0
22	56.5	56.6	-0.1
23	36.3	36.2	0.1
24	25.6	25.6	0.0
25	11.3	11.3	0.0
26	14.5	14.5	0.0
27	172.1	172.1	0.0
28	51.5	51.5	0.0
29	39.6	overlap	-
30	24.3	24.3	0.0
31	22.7	22.7	0.0
32	21.8	21.8	0.0
33	170.4	170.4	0.0
34	60.4	60.4	0.0
35	29.1	29.1	0.0
36	18.5	18.4	0.1
37	18.8	18.8	0.0

[a]All chemical shifts are reported in ppm. Coupling constants are given in parentheses and reported in Hz. All spectra were measured in DMSO-d_6 and referenced to the residual solvent peak at δ_H = 2.50 ppm and δ_C = 39.52 ppm as done in the original isolation report.[2] ^{1}H-NMR spectrum was recorded at 500 MHz. ^{13}C-NMR spectrum was recorded at 126 MHz.

Figure S2. Comparison of (**a**) ^{1}H NMR (DMSO-d_6, 500 MHz), (**b**) ^{13}C NMR (DMSO-d_6, 126 MHz) spectra of synthetic nicrophorusamide A (blue) and nicrophorusamide A (black, screenshot from supporting information of nicrophorusamide A's isolation article published in 2017[2]).

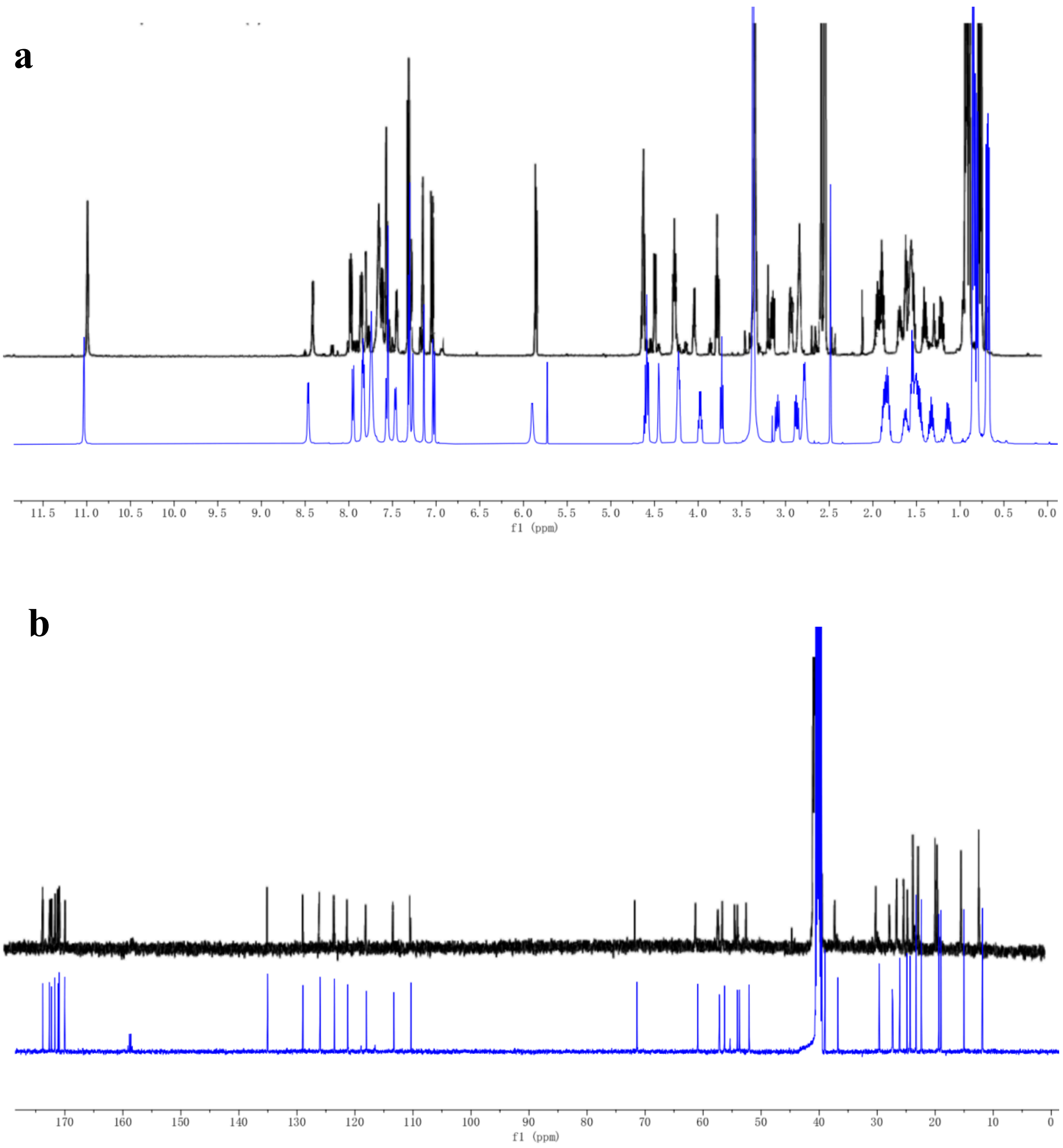

5. NMR Spectra.

^{1}H NMR spectra for 11 (MeOD)

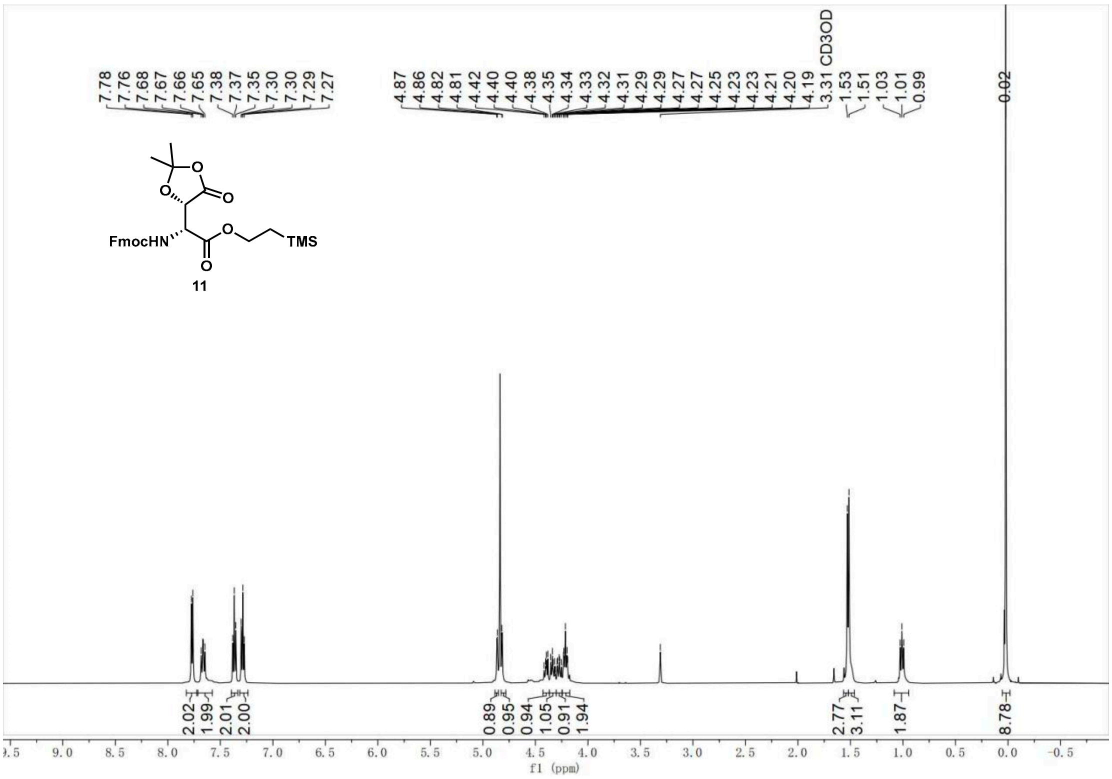

^{13}C NMR spectra for 11 (MeOD)

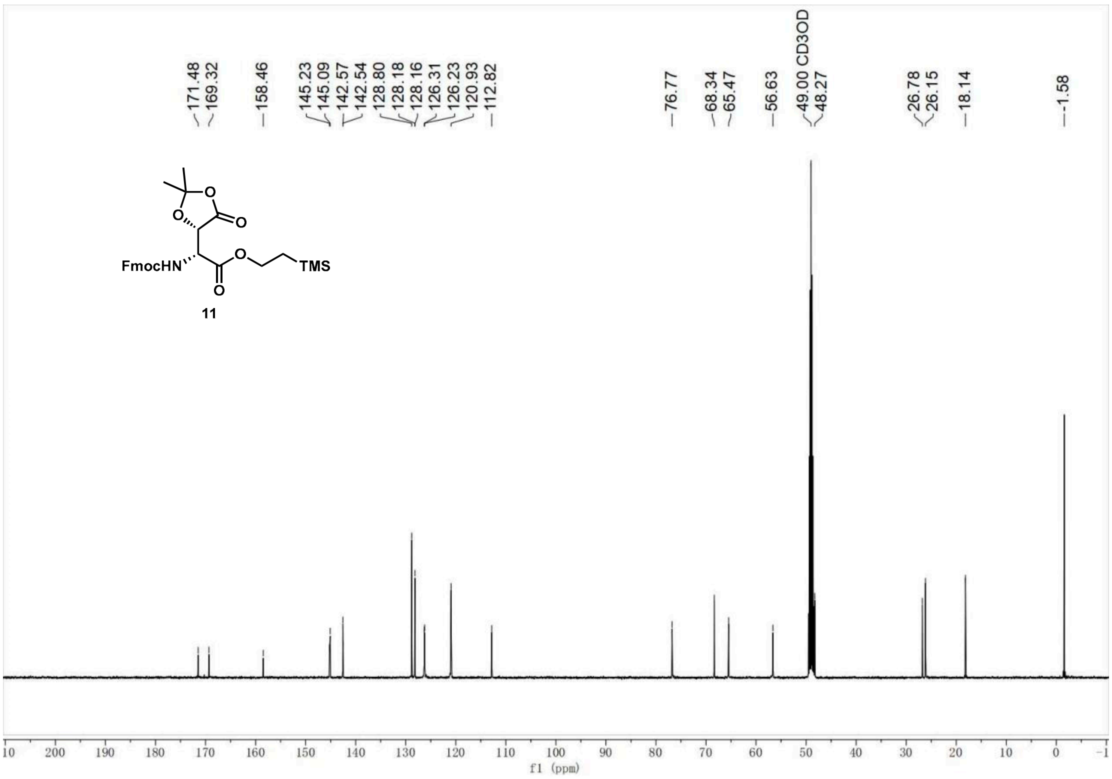

^{1}H NMR spectra for 4 (MeOD)

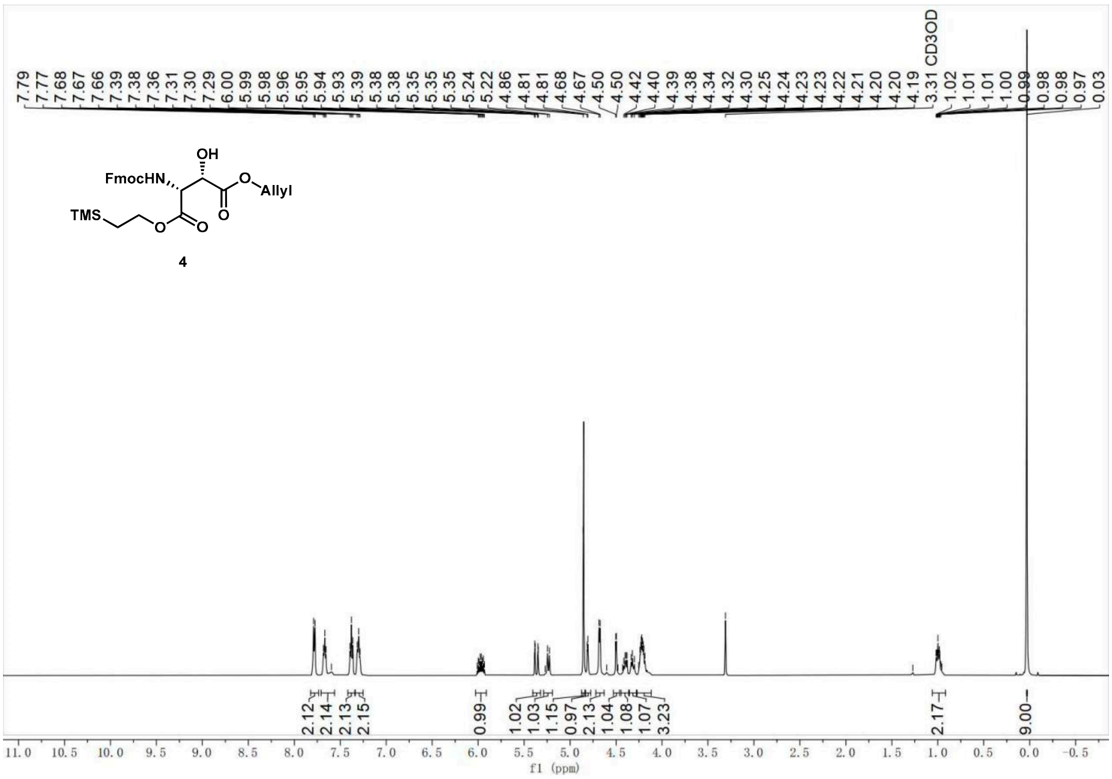

^{13}C NMR spectra for 4 (MeOD)

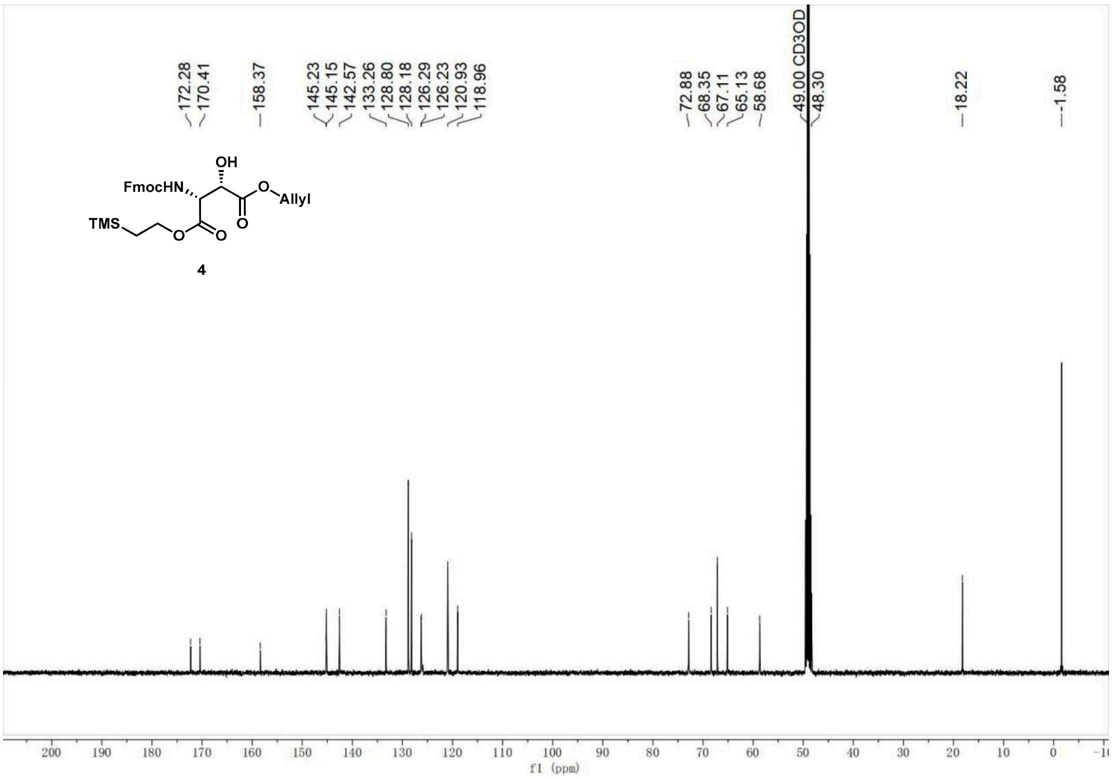

^{1}H NMR spectra for 13 (MeOD)

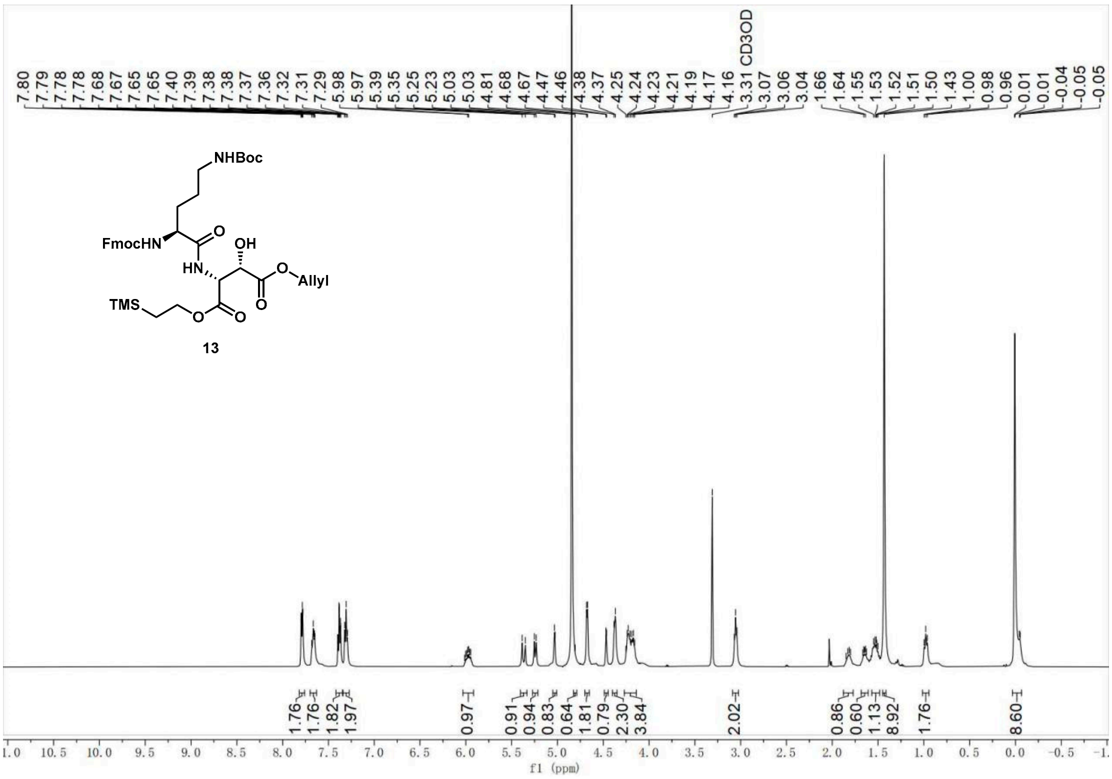

^{13}C NMR spectra for 13 (MeOD)

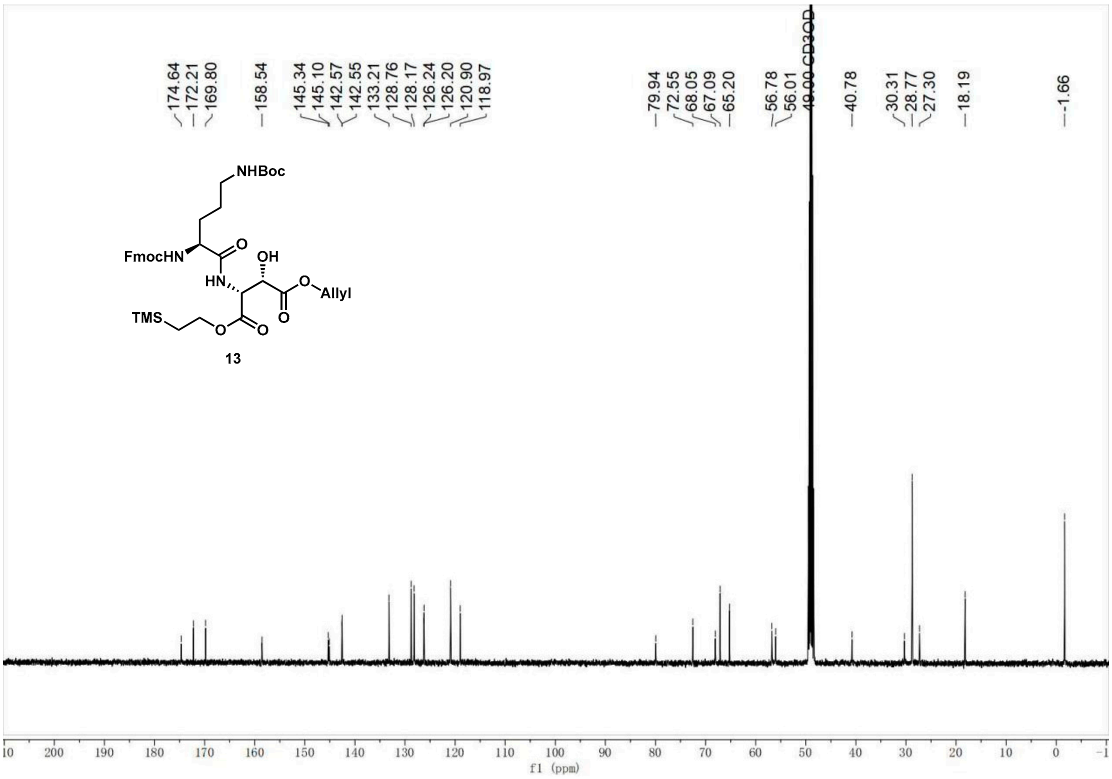

^{1}H NMR spectra for 2 (MeOD)

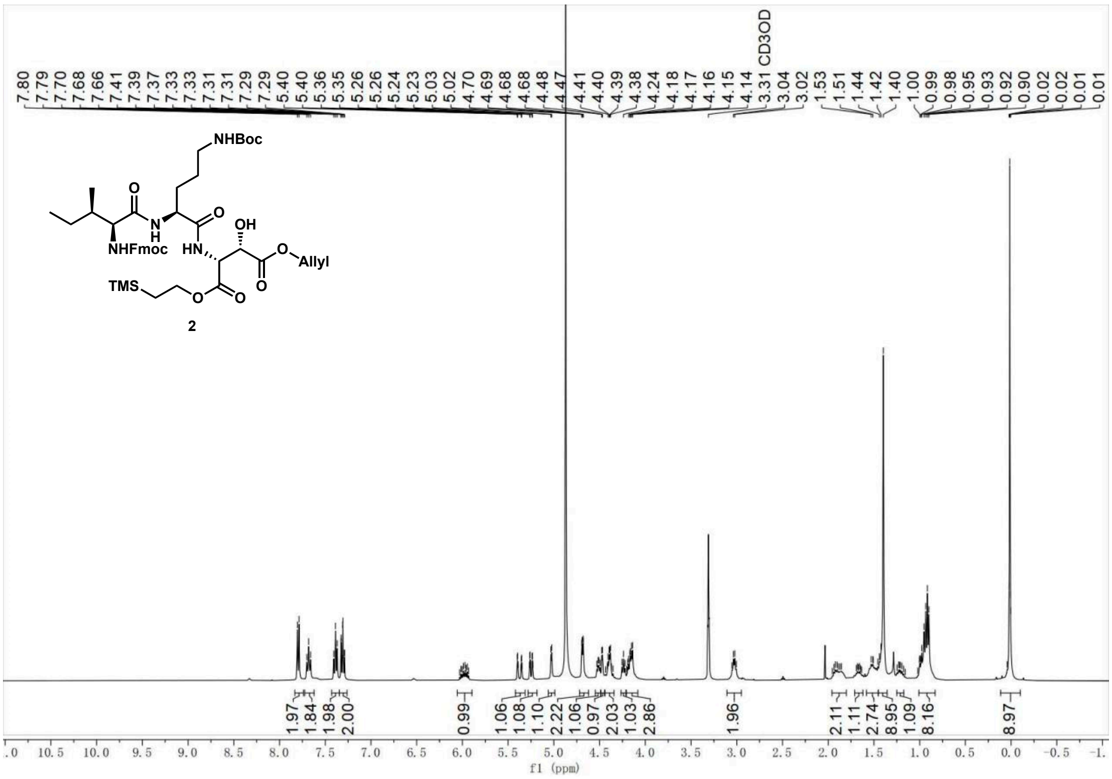

^{13}C NMR spectra for 2 (MeOD)

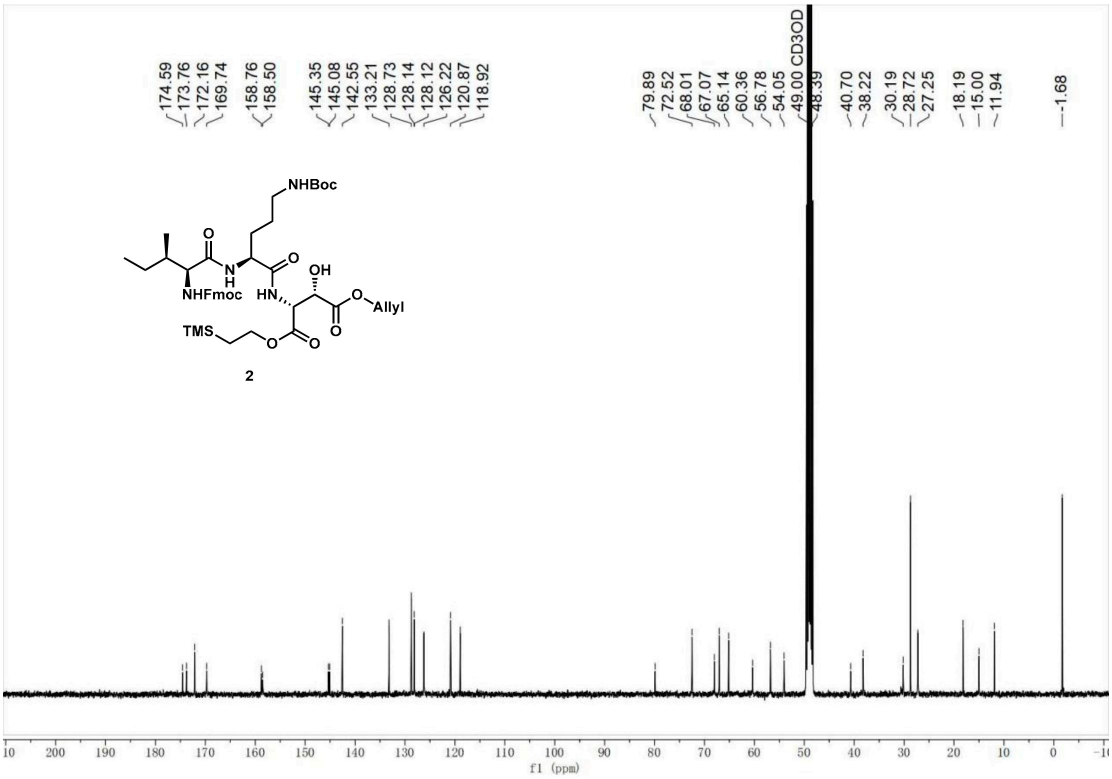

^{1}H NMR spectra for 16 (MeOD)

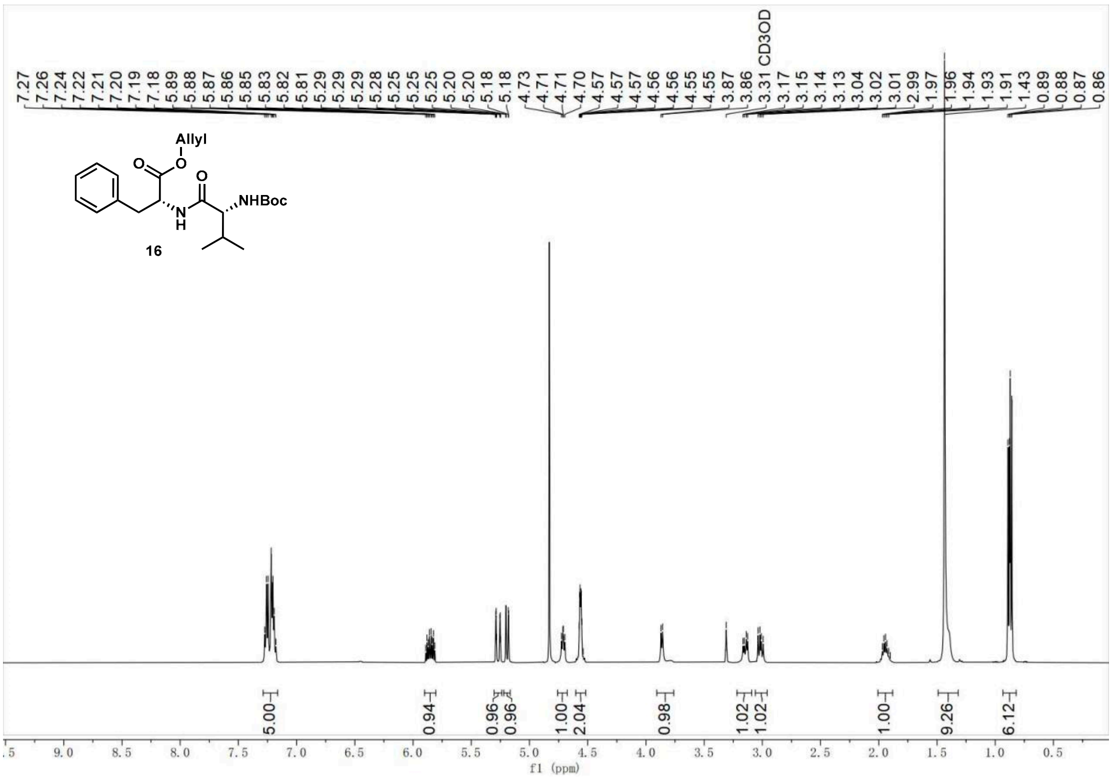

^{13}C NMR spectra for 16 (MeOD)

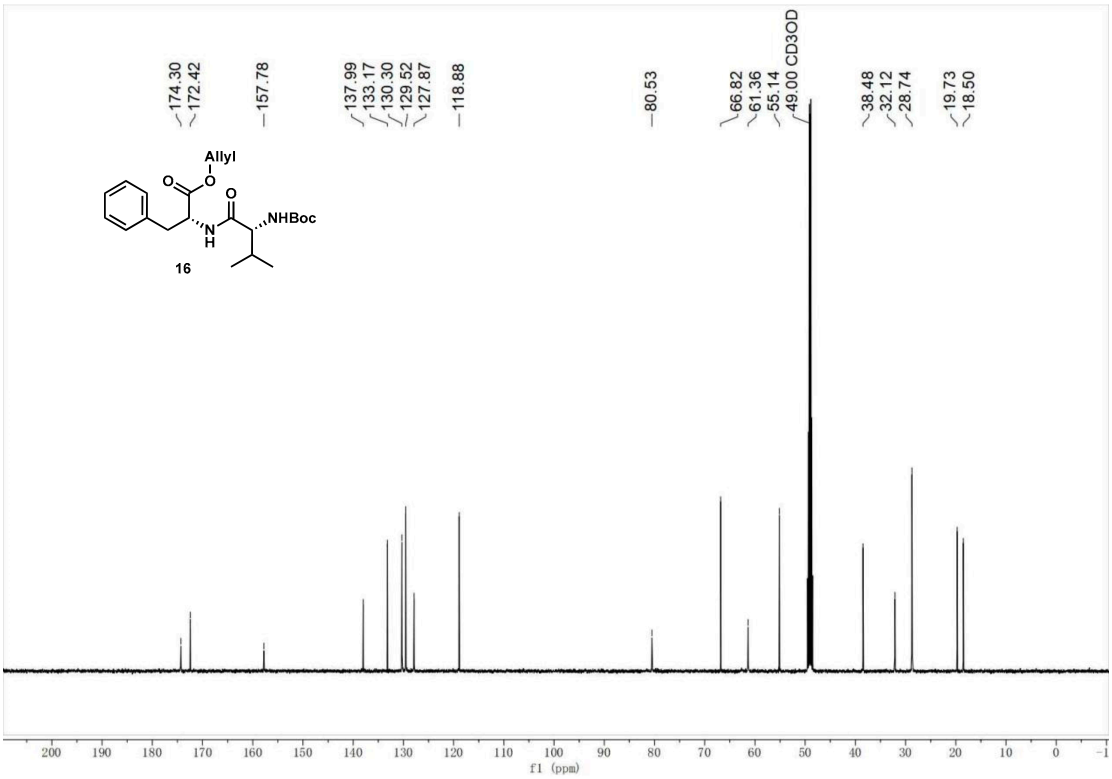

^{1}H NMR spectra for 15 (DMSO-d_6)

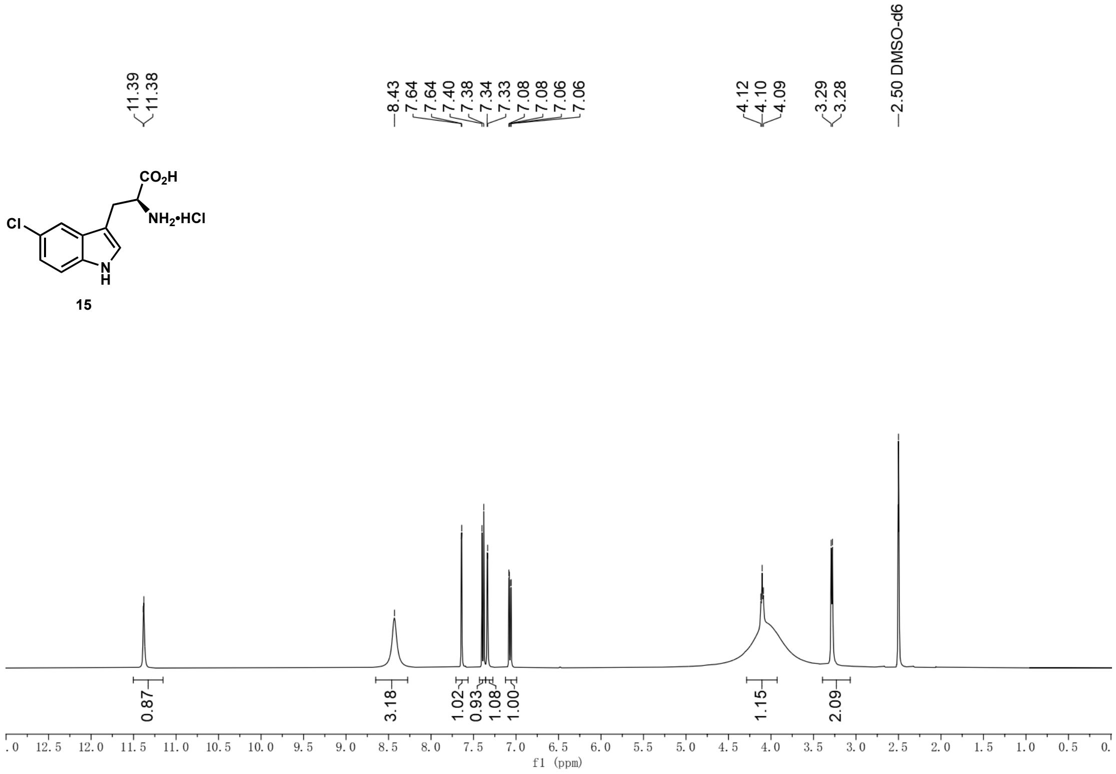

^{13}C NMR spectra for 15 (DMSO-d_6)

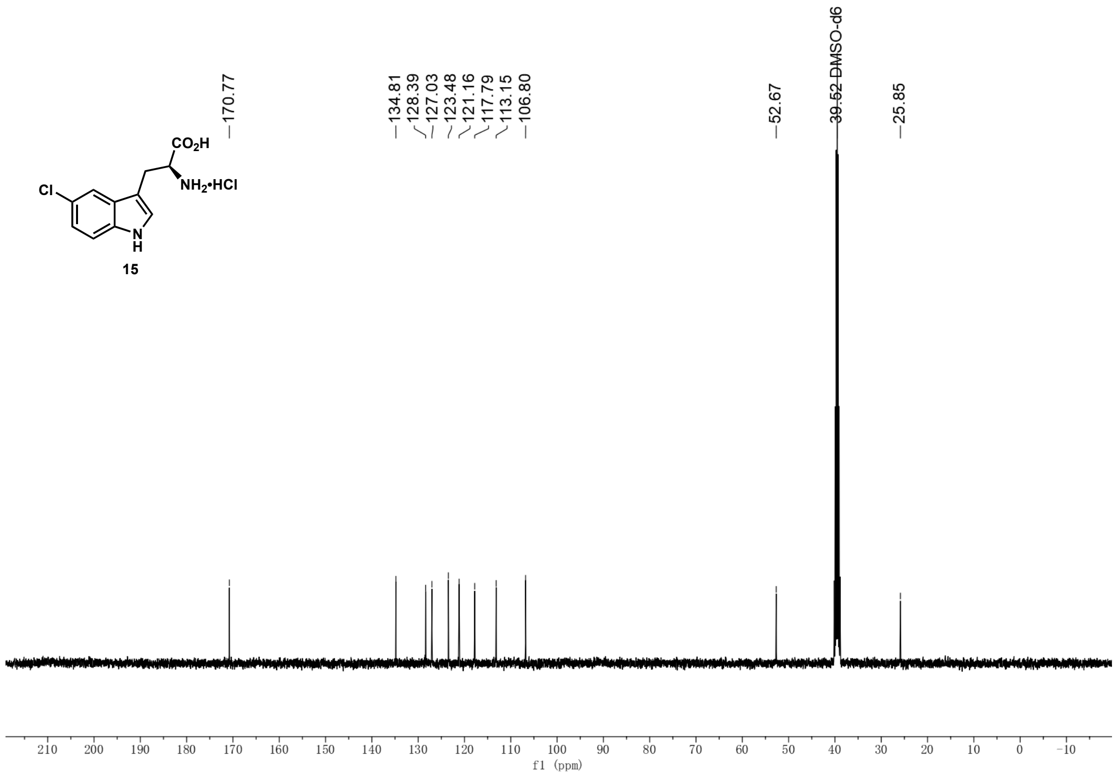

^{1}H NMR spectra for 9 (MeOD)

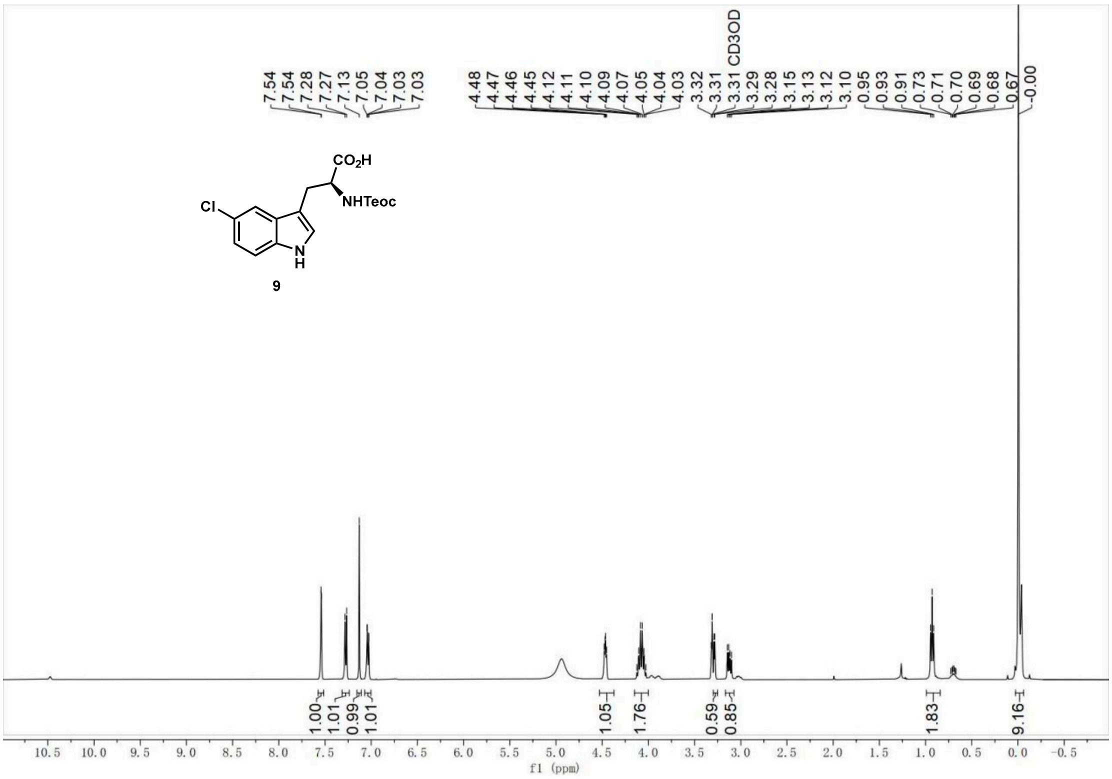

^{13}C NMR spectra for 9 (MeOD)

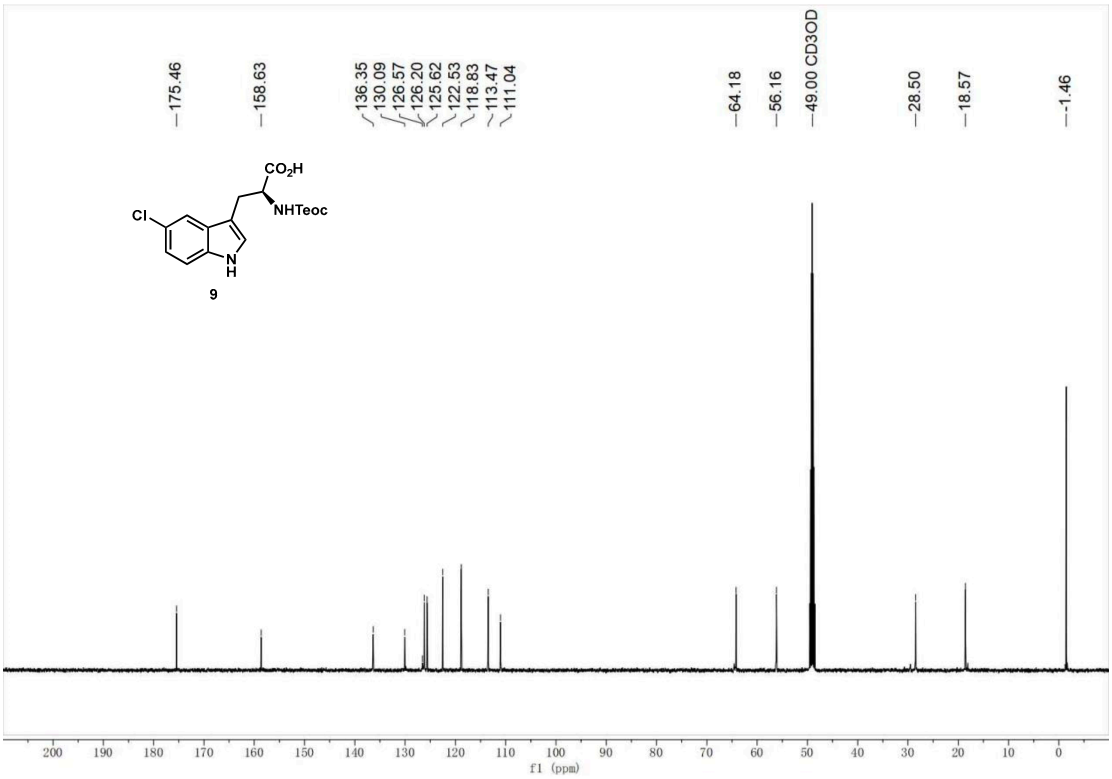

^{1}H NMR spectra for 3 (MeOD)

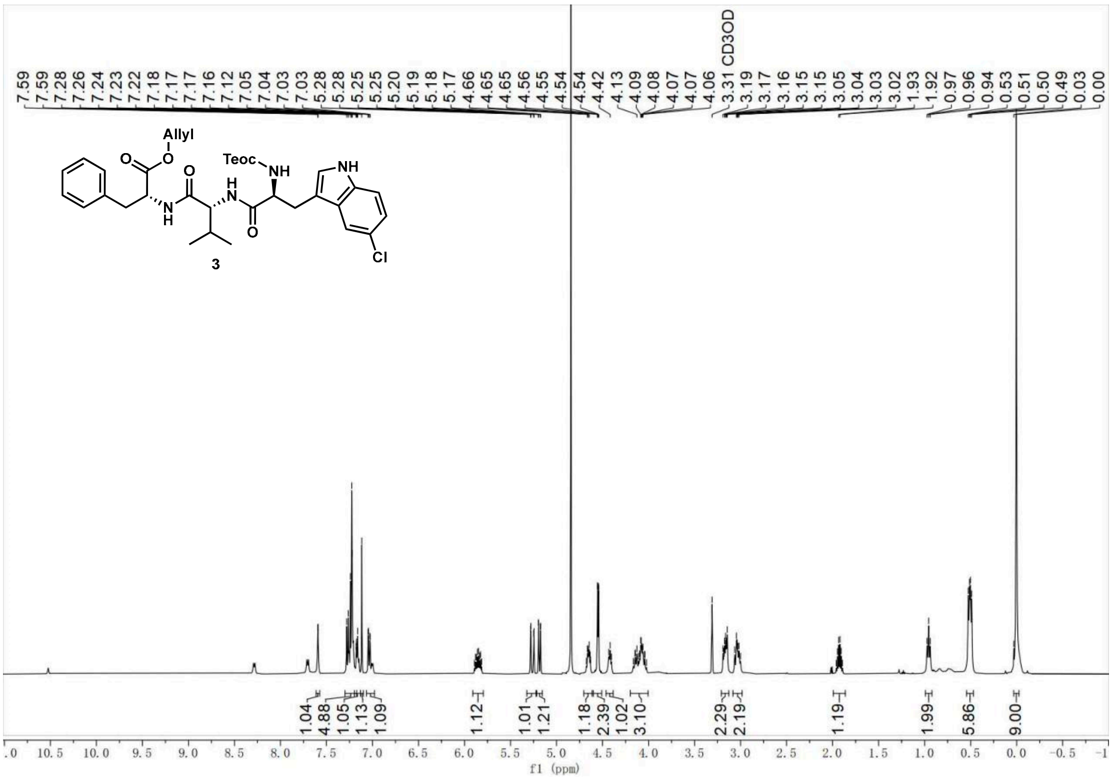

^{13}C NMR spectra for 3 (MeOD)

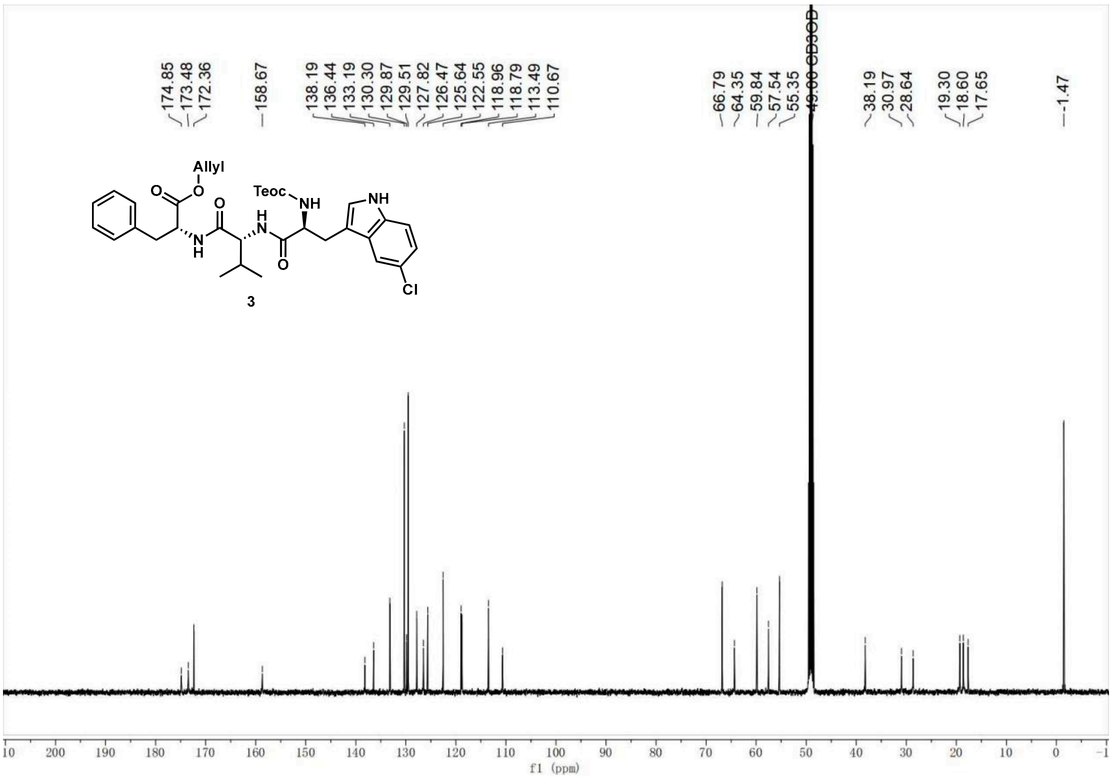

^{1}H NMR spectra for 17 (DMSO-d_6)

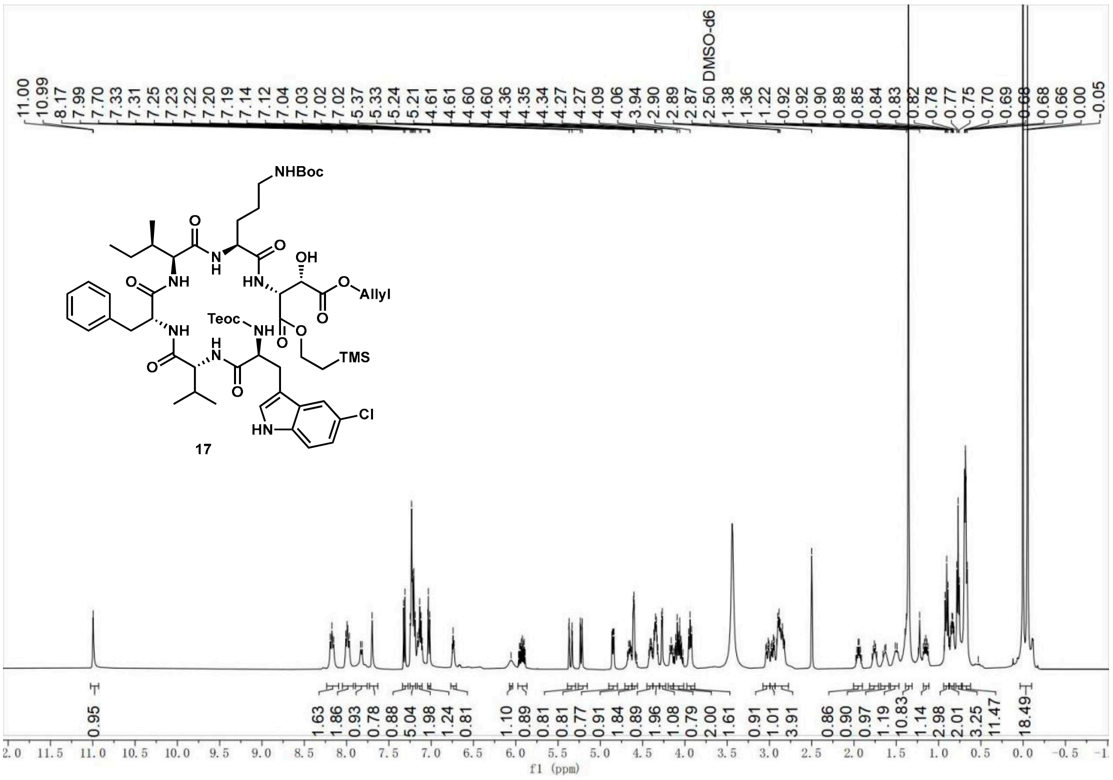

^{13}C NMR spectra for 17 (DMSO-d_6)

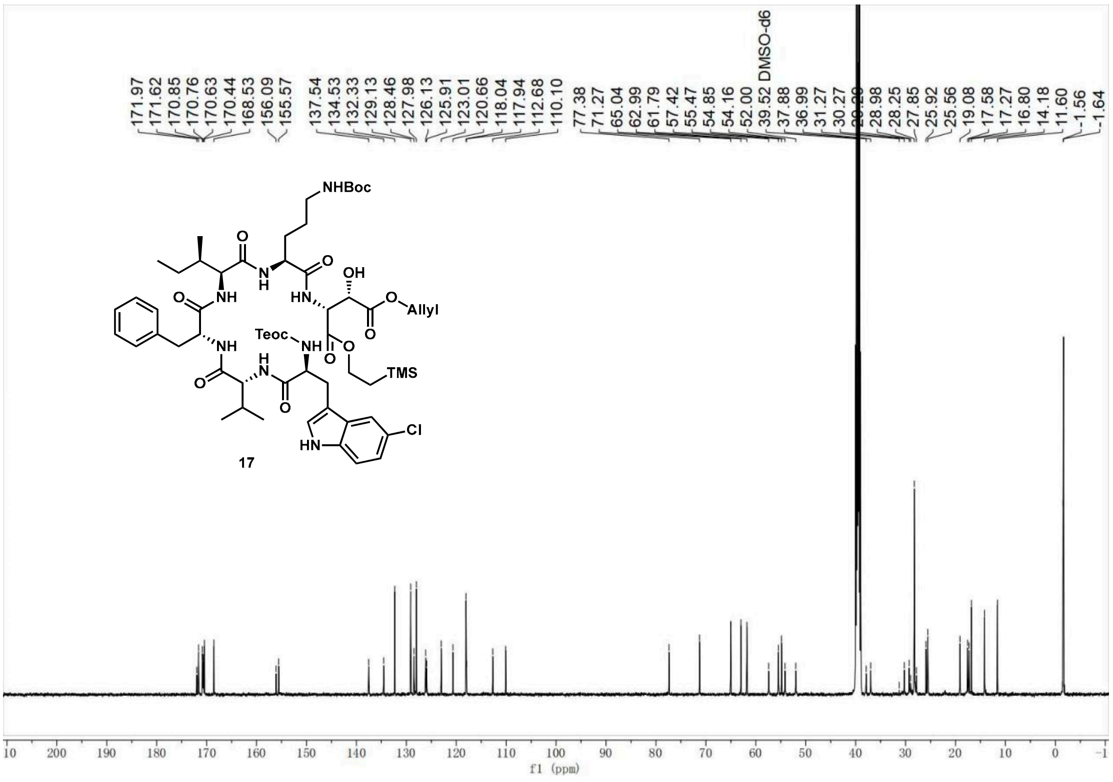

^{1}H NMR spectra for 18 (DMSO-d_6)

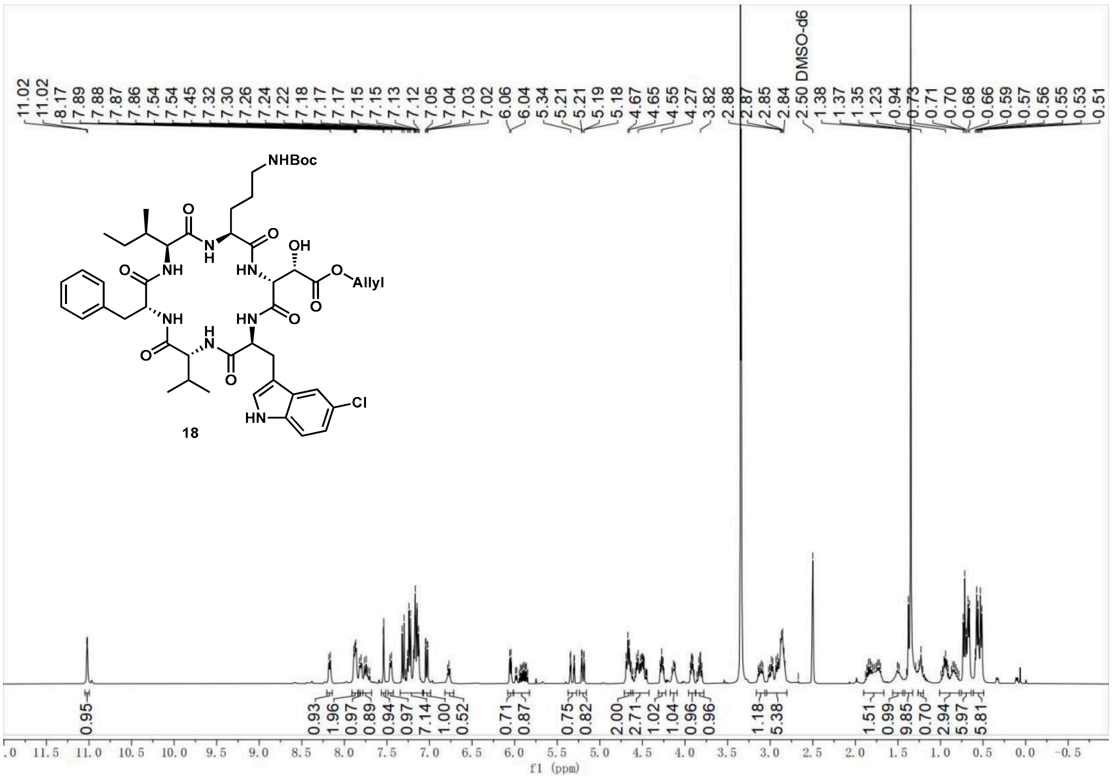

^{13}C NMR spectra for 18 (DMSO-d_6)

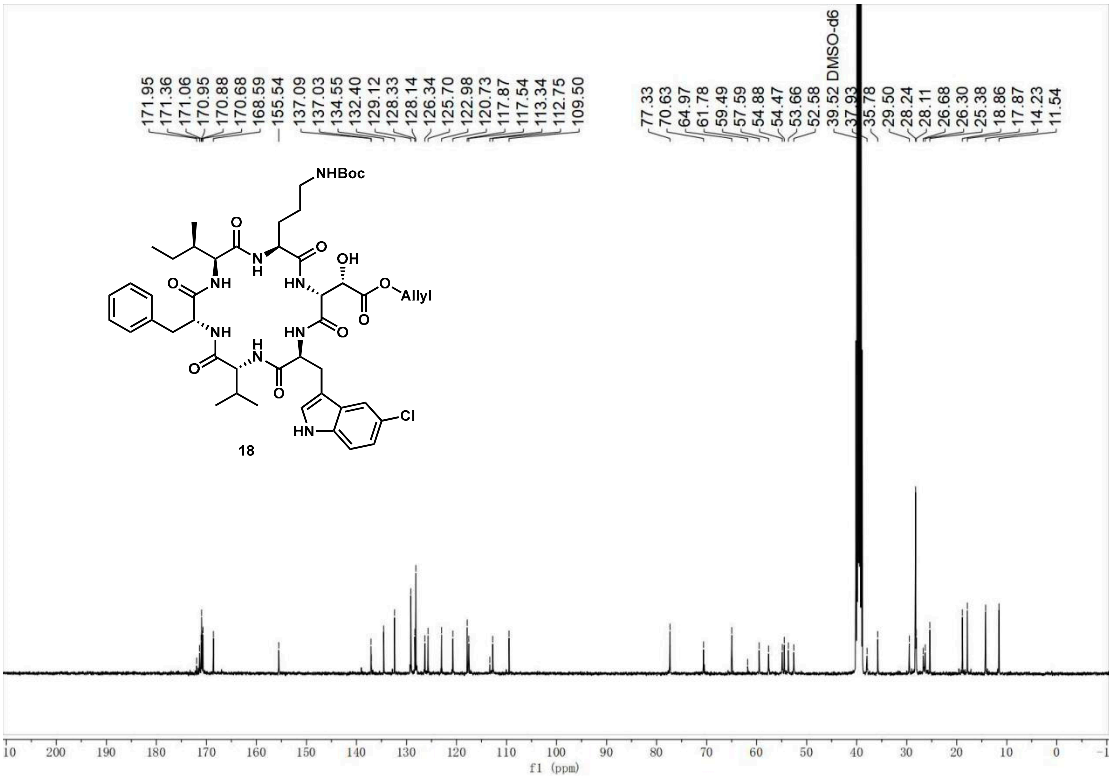

^{1}H NMR spectra for noursamycin A (1) (DMSO-*d₆*)

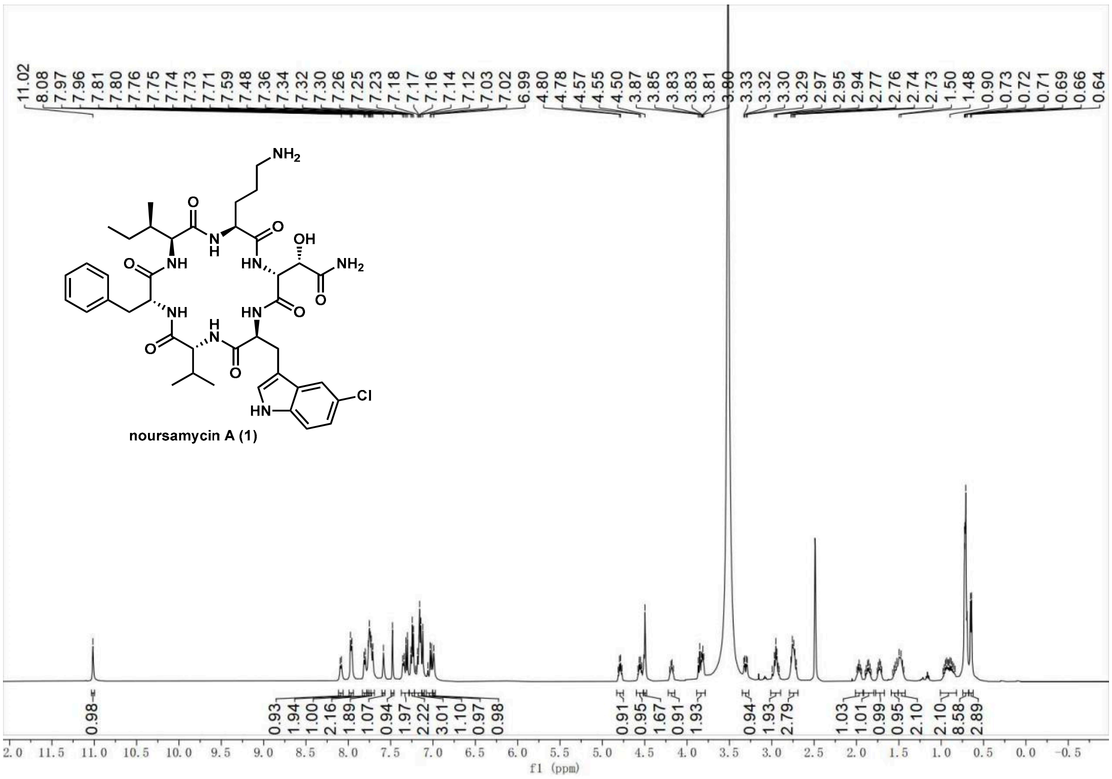

^{13}C NMR spectra for noursamycin A (1) (DMSO-*d₆*)

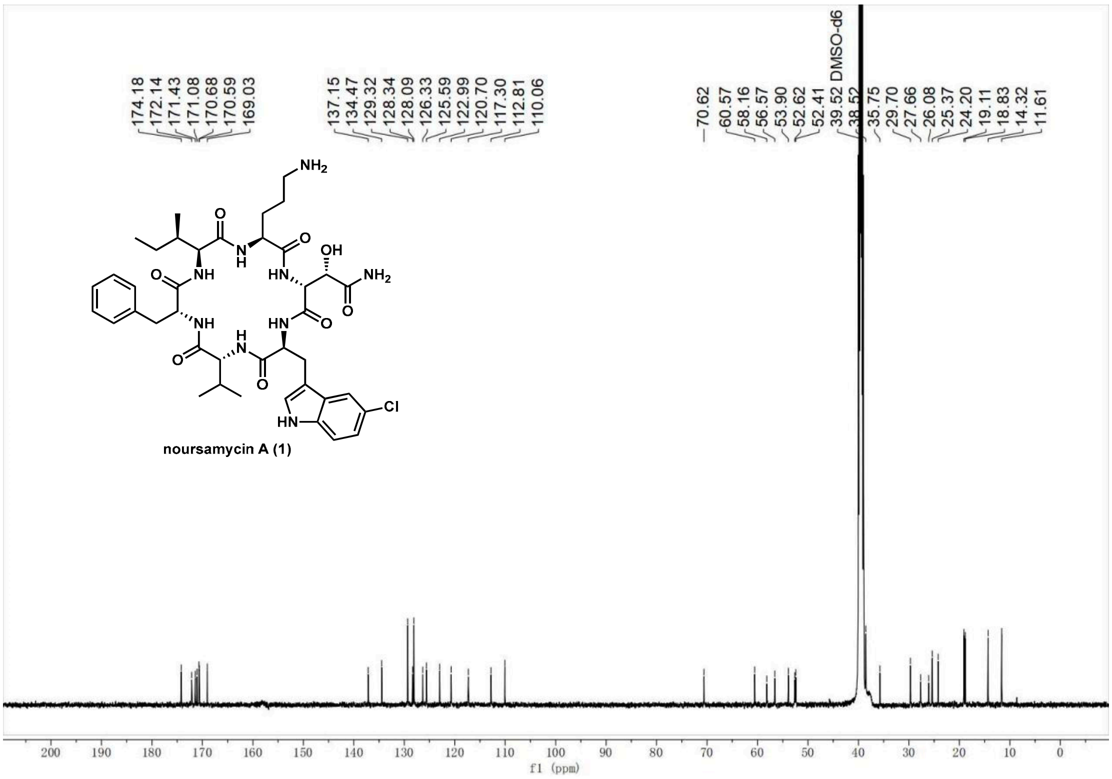

^{1}H NMR spectra for S2 (MeOD)

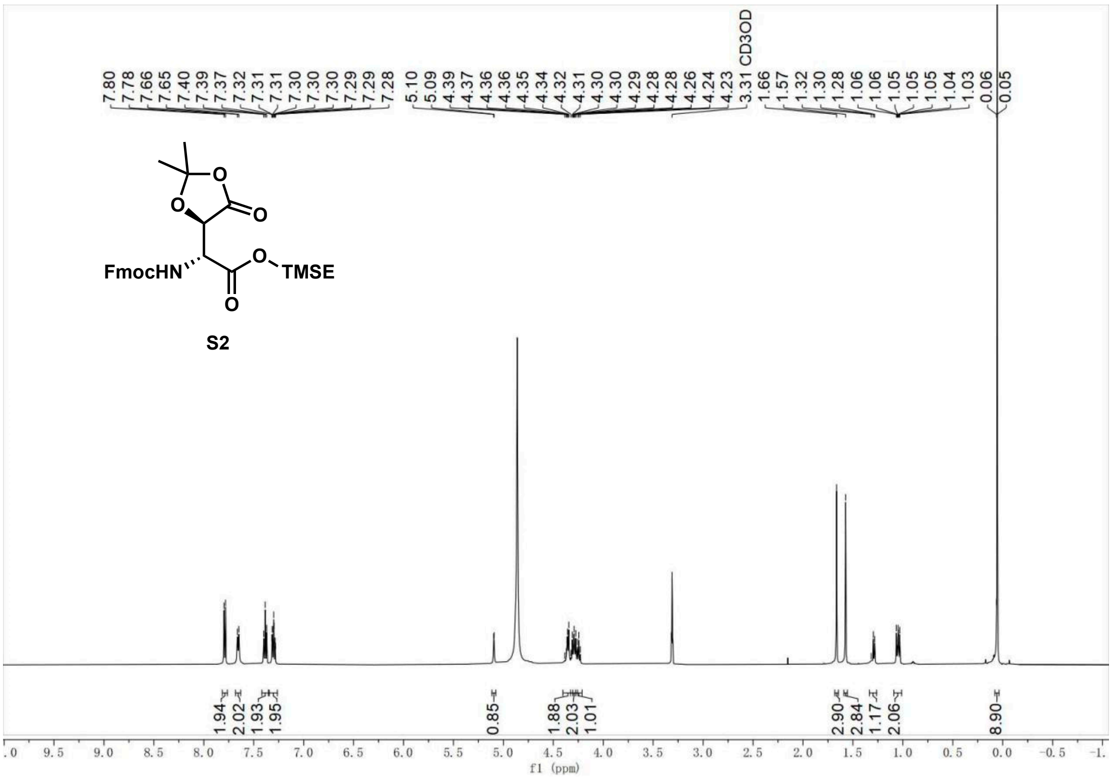

^{13}C NMR spectra for S2 (MeOD)

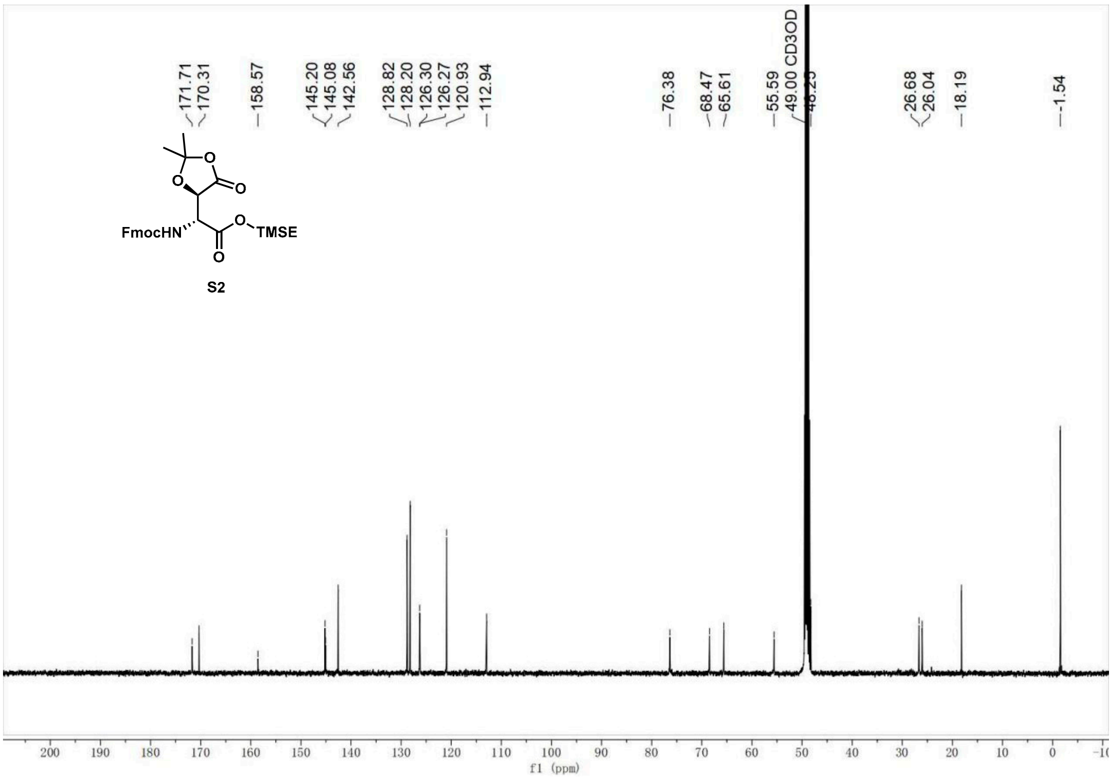

^{1}H NMR spectra for 20 (MeOD)

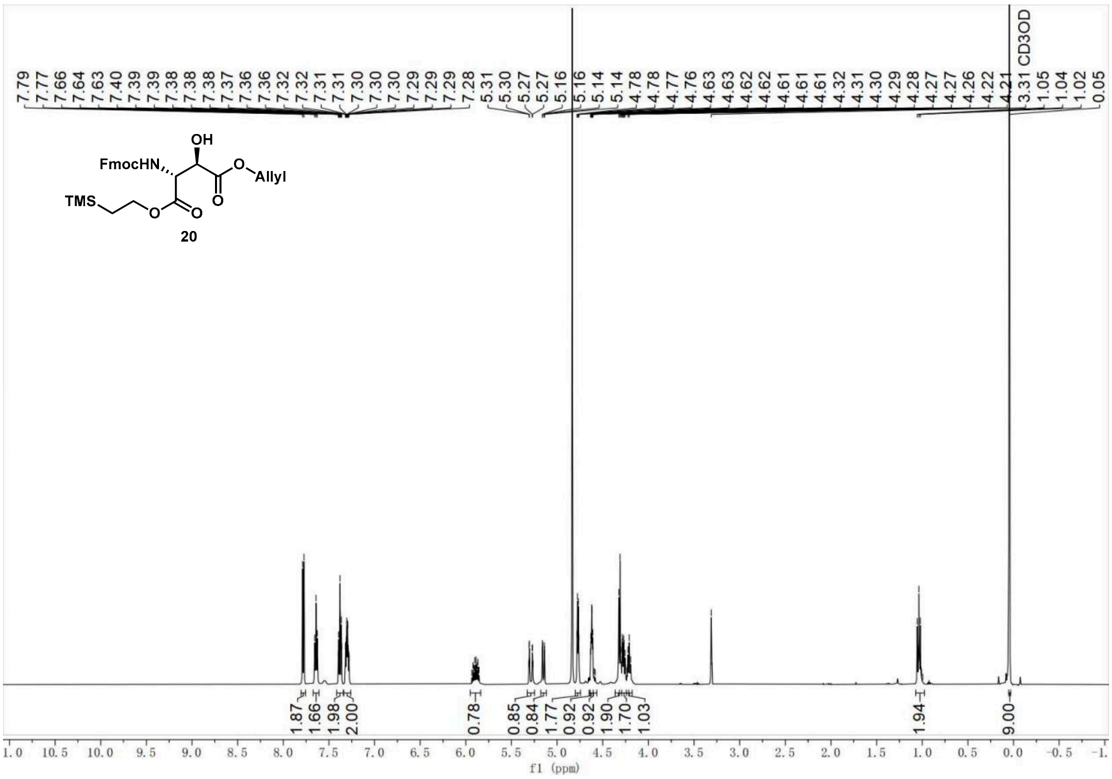

^{13}C NMR spectra for 20 (MeOD)

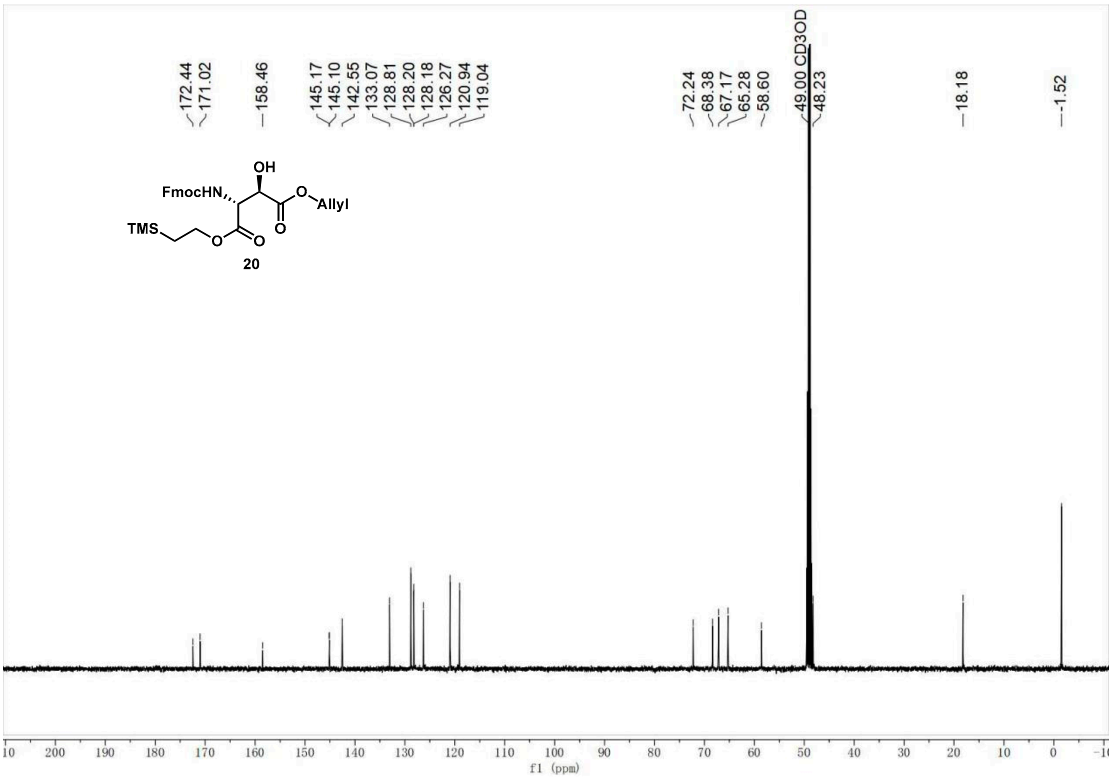

^{1}H NMR spectra for S4 (MeOD)

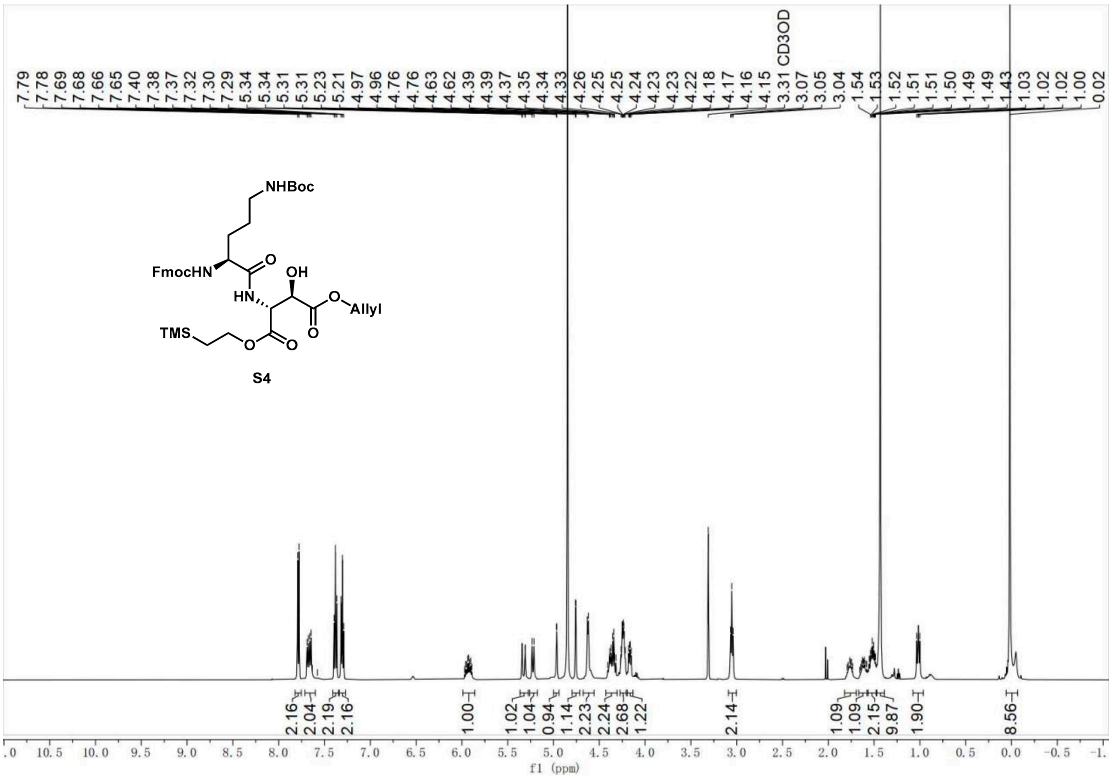

^{13}C NMR spectra for S4 (MeOD)

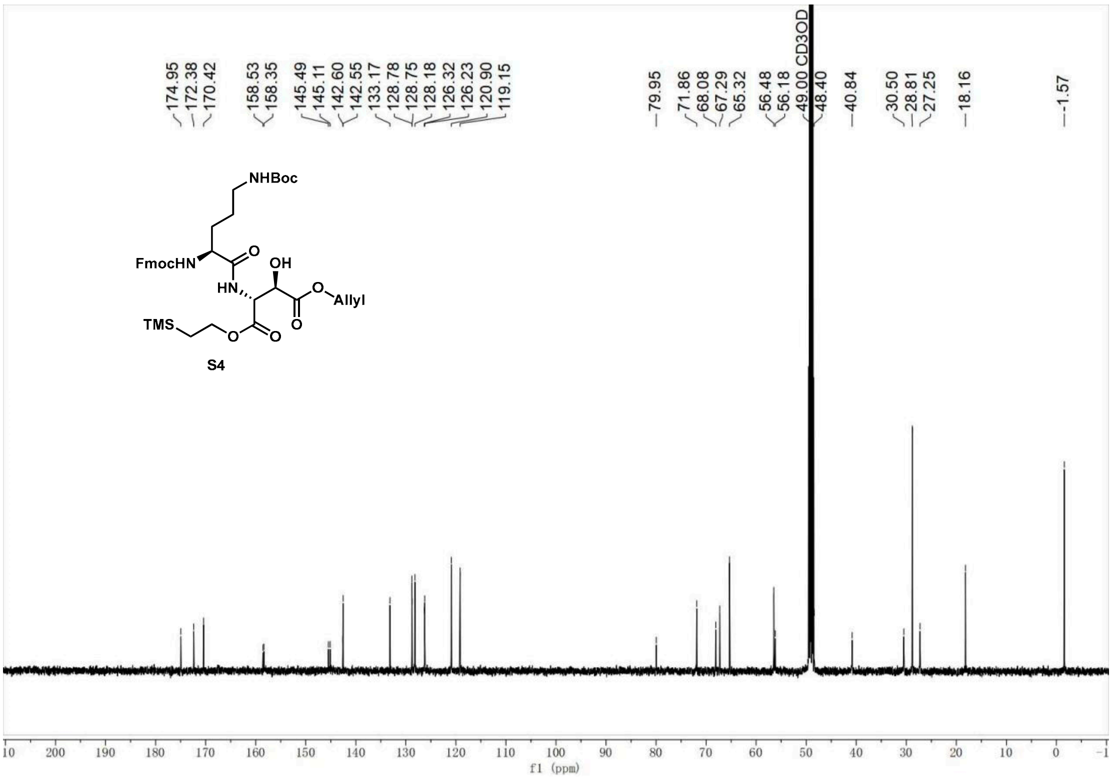

^{1}H NMR spectra for 21 (MeOD)

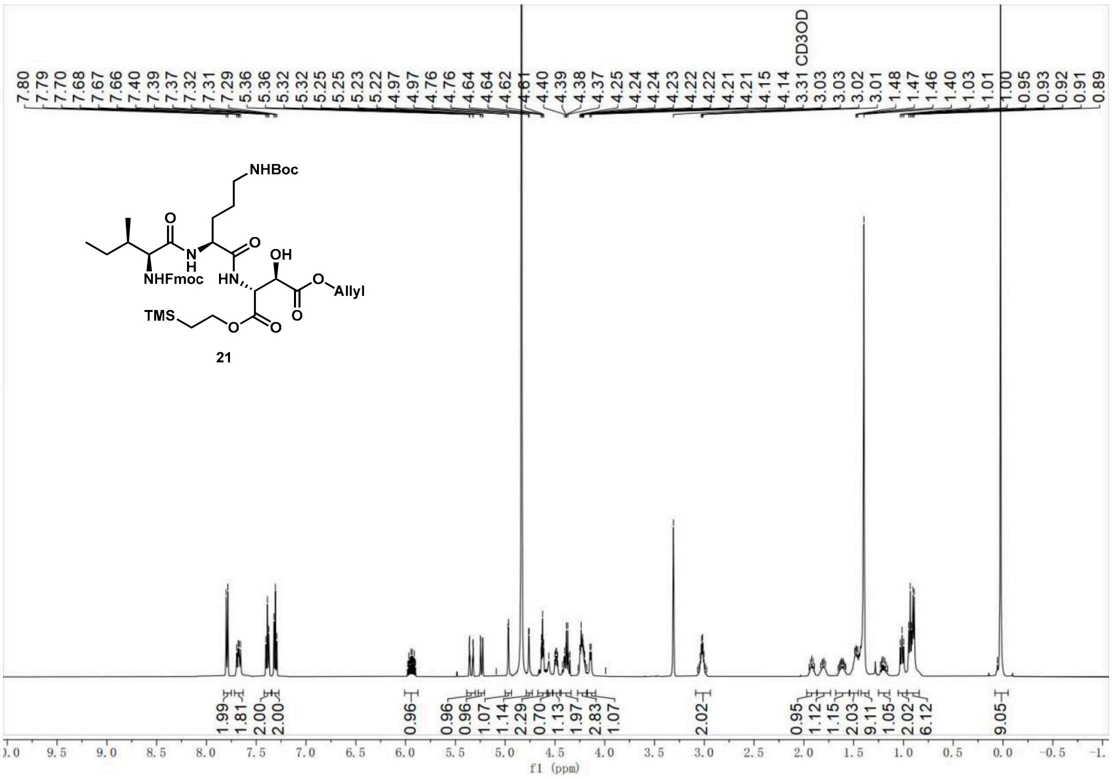

^{13}C NMR spectra for 21 (MeOD)

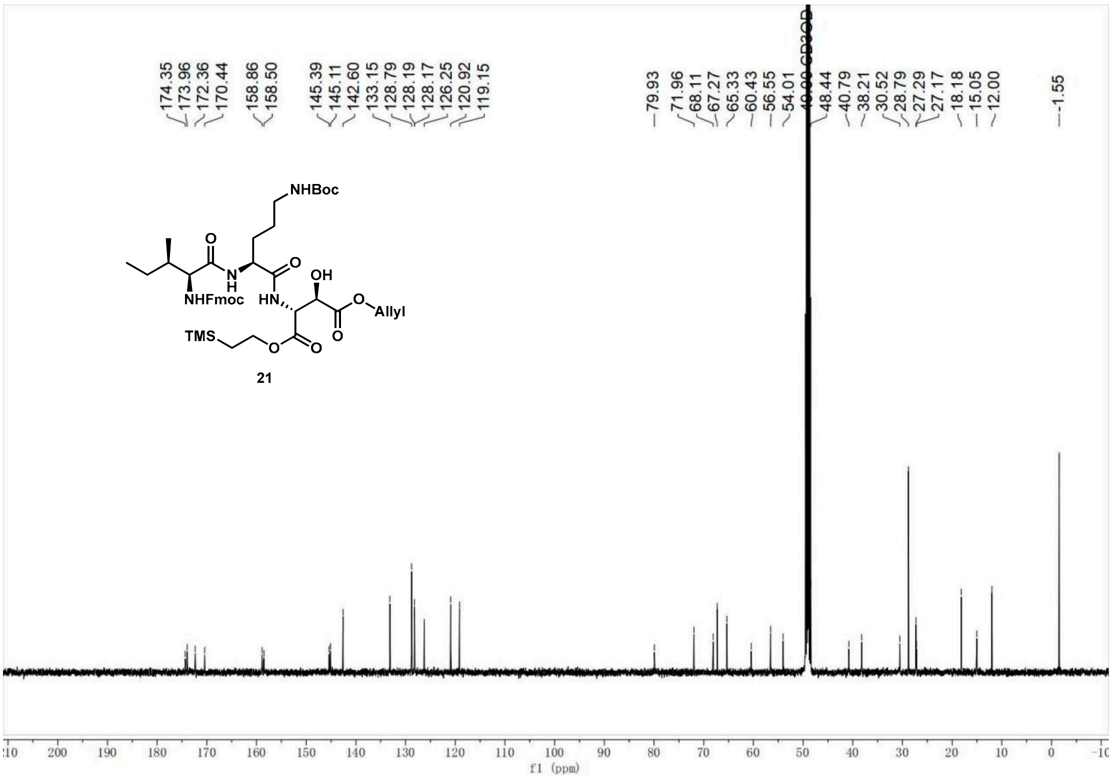

^{1}H NMR spectra for S5 ($CDCl_3$)

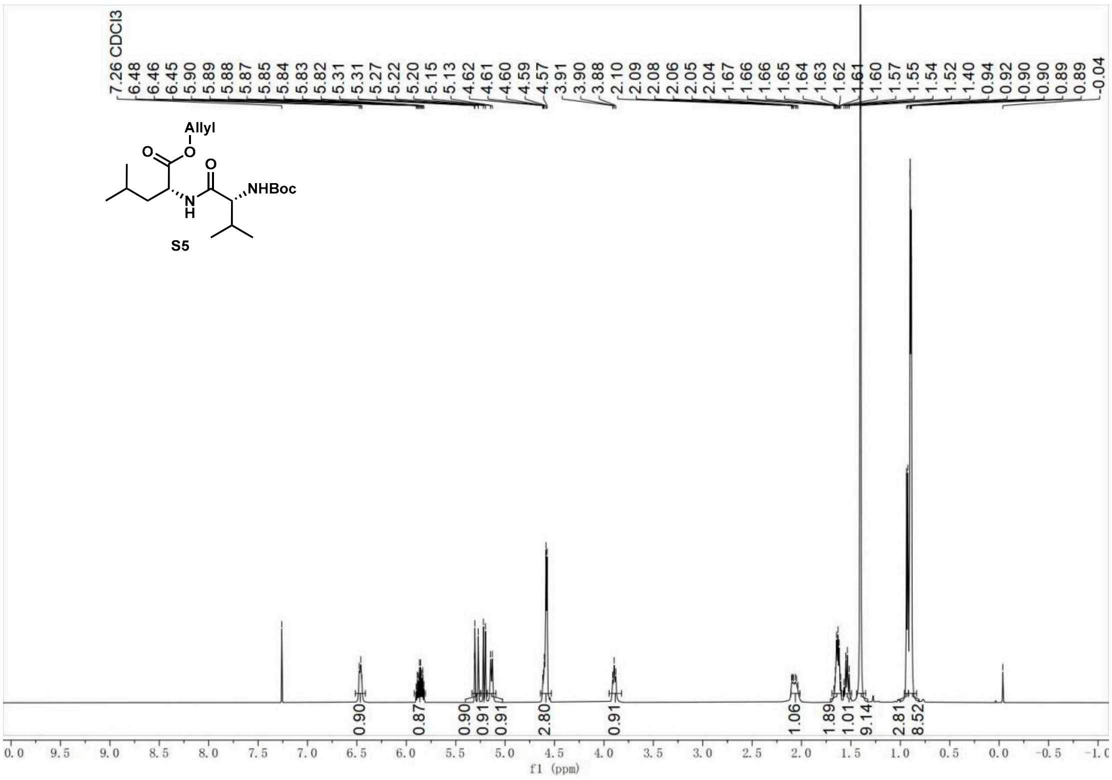

^{13}C NMR spectra for S5 ($CDCl_3$)

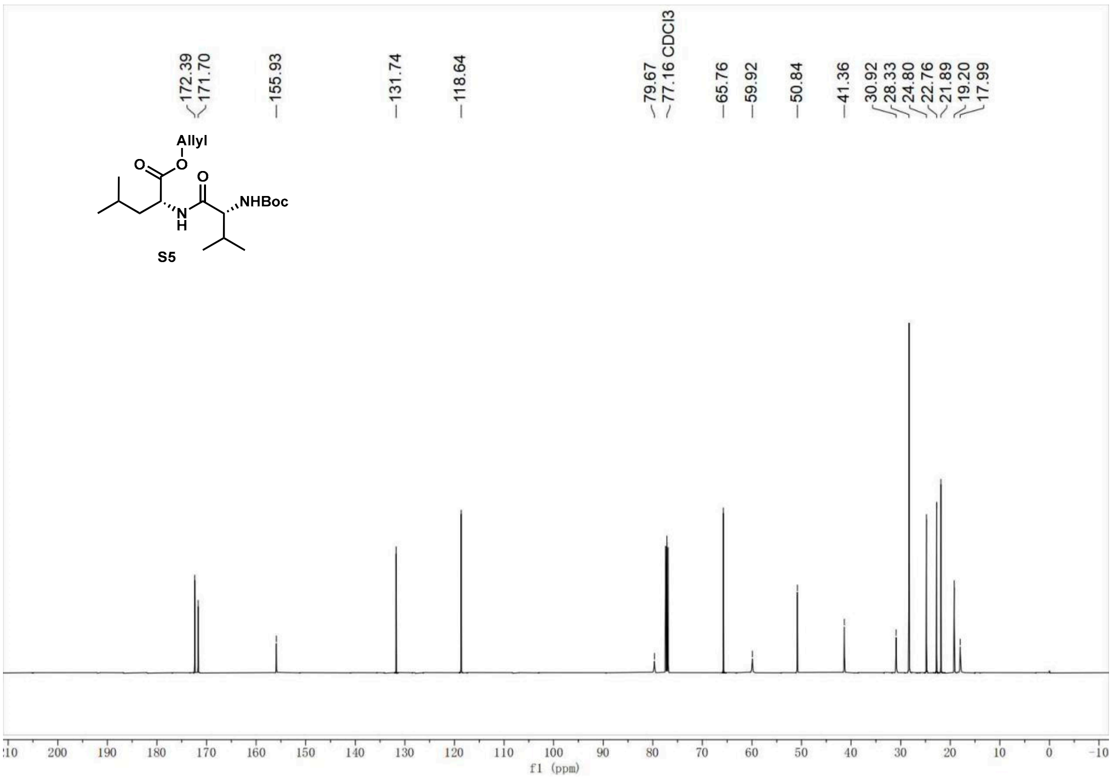

^{1}H NMR spectra for 23 (MeOD)

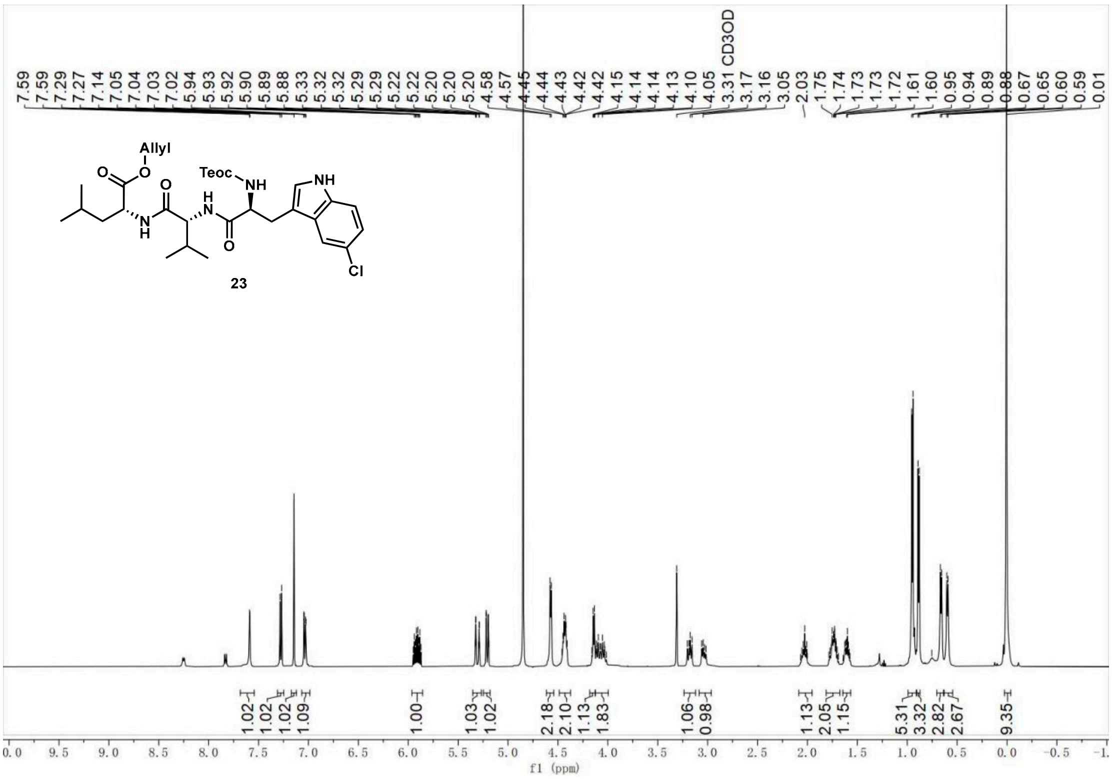

^{13}C NMR spectra for 23 (MeOD)

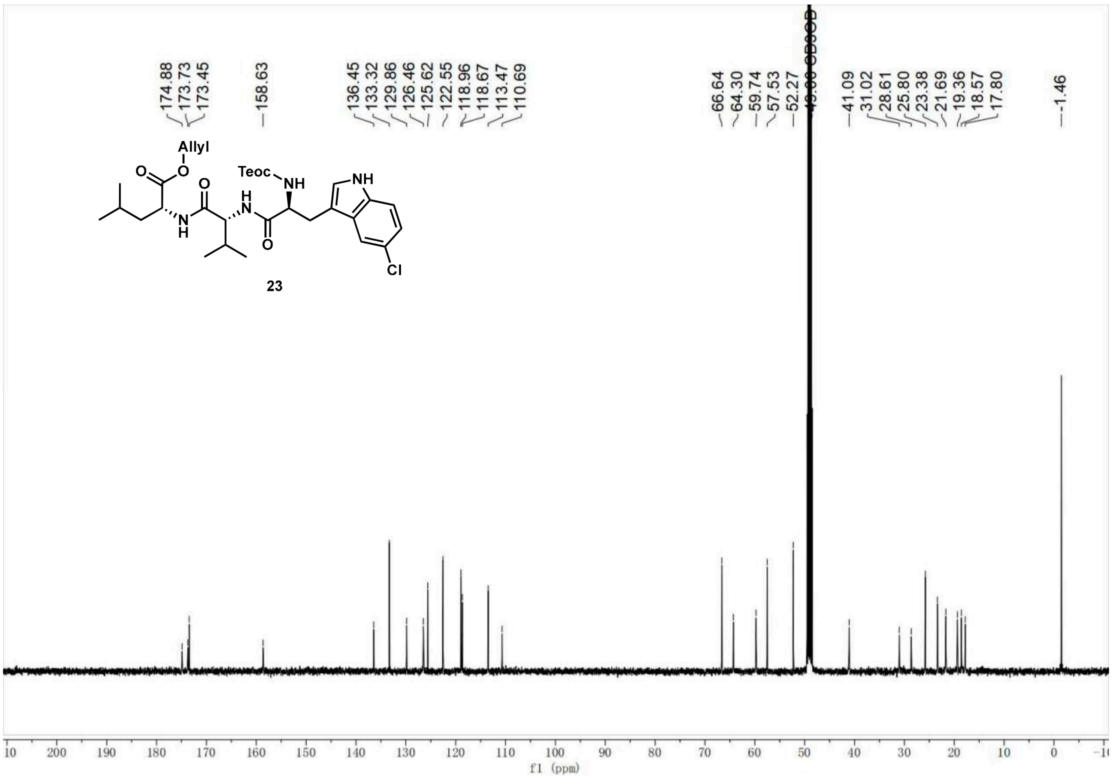

^{1}H NMR spectra for 24 (DMSO-d_6)

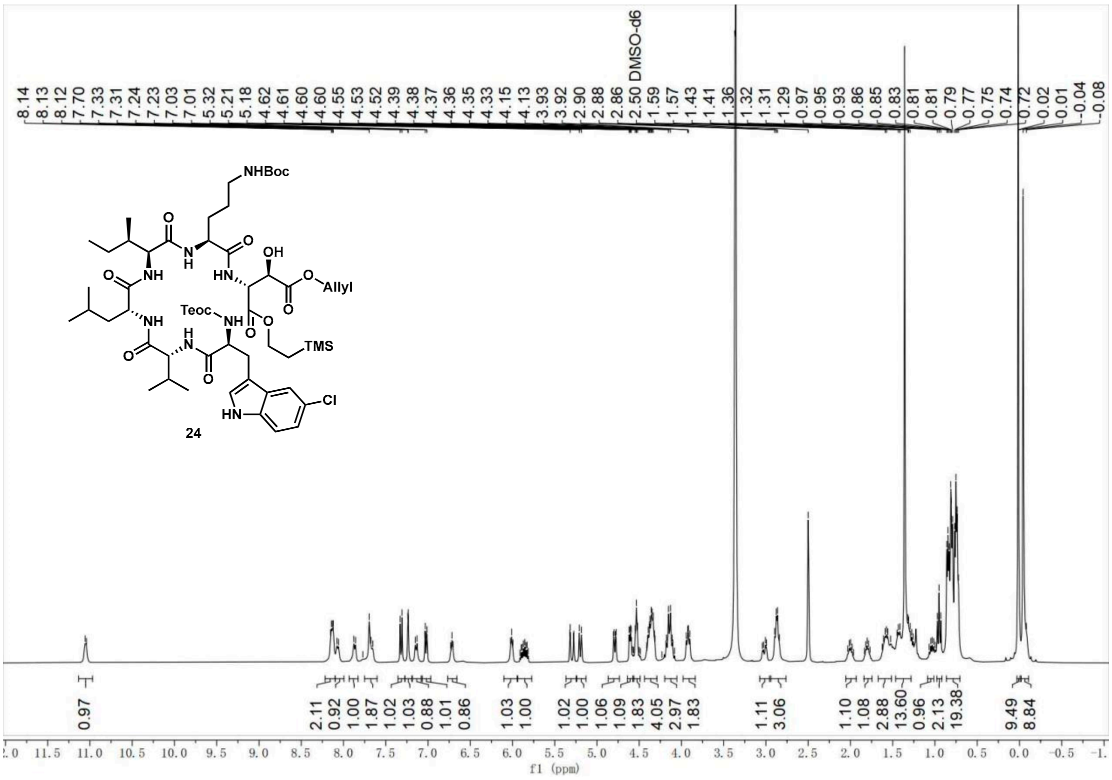

^{13}C NMR spectra for 24 (DMSO-d_6)

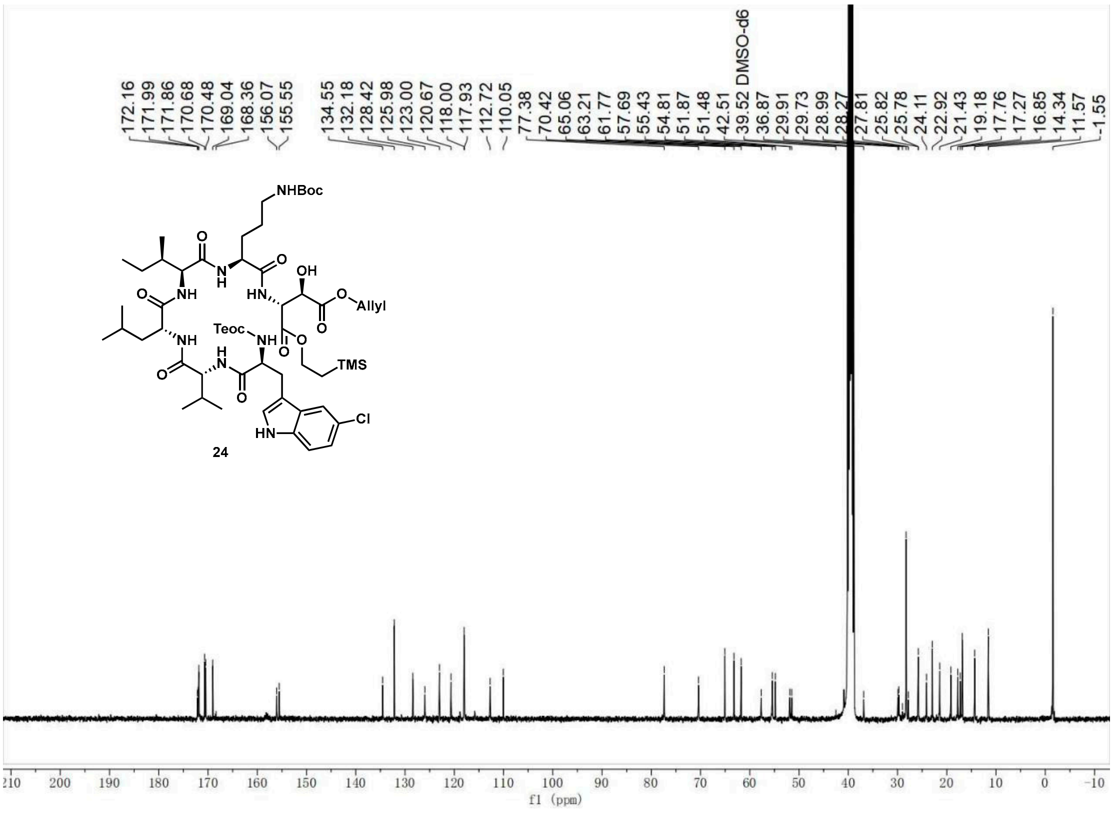

^{1}H NMR spectra for S9 (DMSO-d_6)

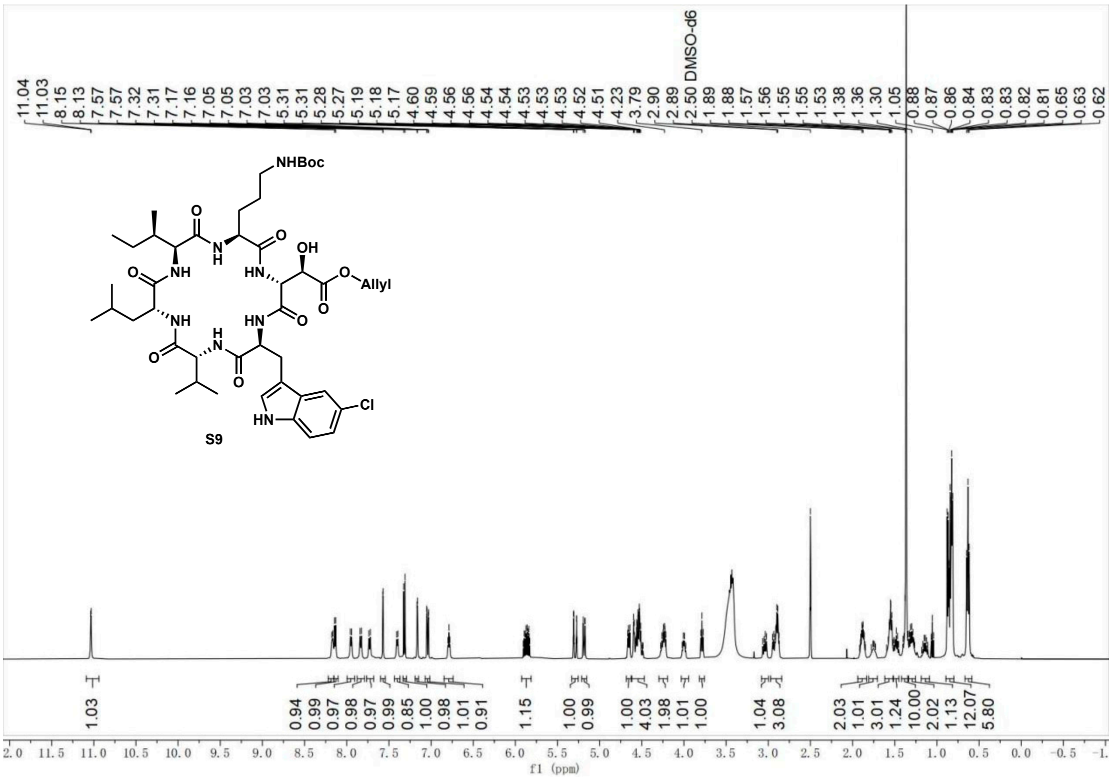

^{13}C NMR spectra for S9 (DMSO-d_6)

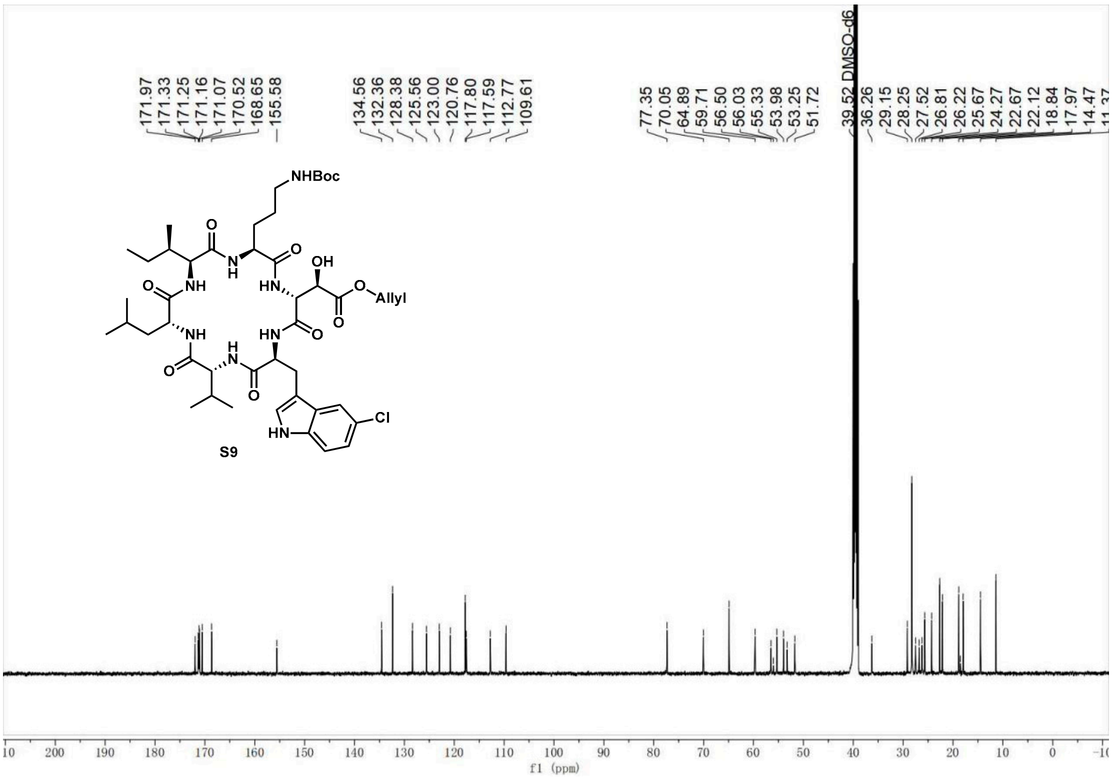

^{1}H NMR spectra for nicrophorusamide A (25) (DMSO-d_6)

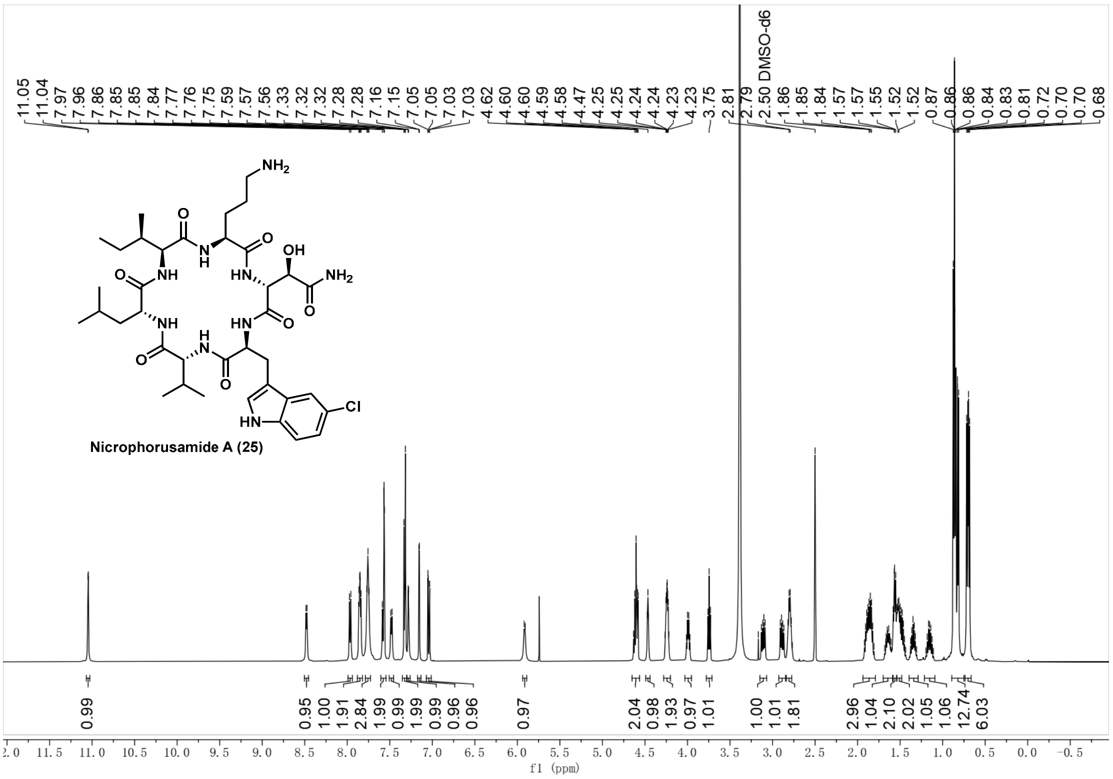

^{13}C NMR spectra for nicrophorusamide A (25) (DMSO-d_6)

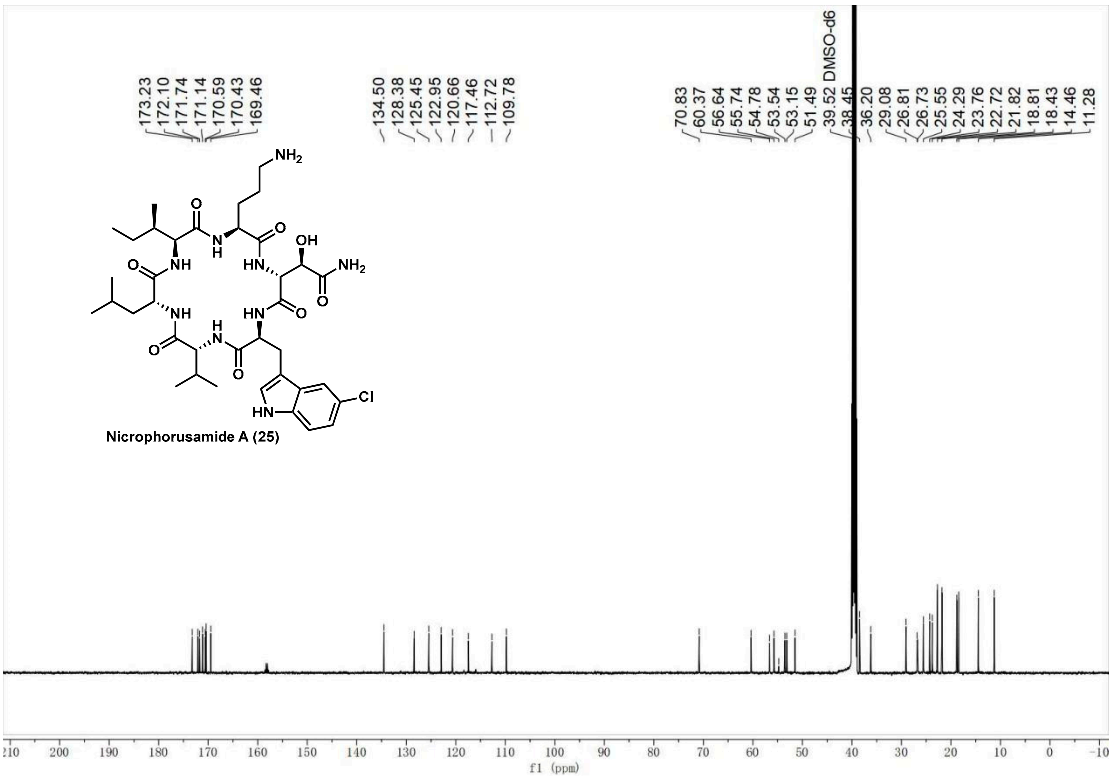

6. References.

(1) Mudalungu, C. M.; von Torne, W. J.; Voigt, K.; Ruckert, C.; Schmitz, S.; Sekurova, O. N.; Zotchev, S. B.; Sussmuth, R. D. Noursamycins, Chlorinated Cyclohexapeptides Identified from Molecular Networking of Streptomyces noursei NTR-SR4. *J Nat Prod* **2019**, *82* (6), 1478-1486. DOI: 10.1021/acs.jnatprod.8b00967.

(2) Shin, Y. H.; Bae, S.; Sim, J.; Hur, J.; Jo, S. I.; Shin, J.; Suh, Y. G.; Oh, K. B.; Oh, D. C. Nicrophorusamides A and B, Antibacterial Chlorinated Cyclic Peptides from a Gut Bacterium of the Carrion Beetle Nicrophorus concolor. *J Nat Prod* **2017**, *80* (11), 2962-2968. DOI: 10.1021/acs.jnatprod.7b00506.
